# Assignment of structural domains in proteins using diffusion kernels on graphs

**DOI:** 10.1186/s12859-022-04902-9

**Published:** 2022-09-08

**Authors:** Mohammad Taheri-Ledari, Amirali Zandieh, Seyed Peyman Shariatpanahi, Changiz Eslahchi

**Affiliations:** 1 Department of Bioinformatics, Institute of Biochemistry and Biophysics (IBB), University of Tehran, Tehran, Iran; 2 Department of Biophysics, Institute of Biochemistry and Biophysics (IBB), University of Tehran, Tehran, Iran; 3 Department of Computer and Data Sciences, Faculty of Mathematical Sciences, Shahid Beheshti University, Tehran, Iran; 4 School of Biological Sciences, Institute for Research in Fundamental Sciences (IPM), Tehran, Iran

**Keywords:** Protein structure, Graph node kernel, Protein domain assignment, Clustering, Diffusion kernel

## Abstract

Though proposing algorithmic approaches for protein domain decomposition has been of high interest, the inherent ambiguity to the problem makes it still an active area of research. Besides, accurate automated methods are in high demand as the number of solved structures for complex proteins is on the rise. While majority of the previous efforts for decomposition of 3D structures are centered on the developing clustering algorithms, employing enhanced measures of proximity between the amino acids has remained rather uncharted. If there exists a kernel function that in its reproducing kernel Hilbert space, structural domains of proteins become well separated, then protein structures can be parsed into domains without the need to use a complex clustering algorithm. Inspired by this idea, we developed a protein domain decomposition method based on diffusion kernels on protein graphs. We examined all combinations of four graph node kernels and two clustering algorithms to investigate their capability to decompose protein structures. The proposed method is tested on five of the most commonly used benchmark datasets for protein domain assignment plus a comprehensive non-redundant dataset. The results show a competitive performance of the method utilizing one of the diffusion kernels compared to four of the best automatic methods. Our method is also able to offer alternative partitionings for the same structure which is in line with the subjective definition of protein domain. With a competitive accuracy and balanced performance for the simple and complex structures despite relying on a relatively naive criterion to choose optimal decomposition, the proposed method revealed that diffusion kernels on graphs in particular, and kernel functions in general are promising measures to facilitate parsing proteins into domains and performing different structural analysis on proteins. The size and interconnectedness of the protein graphs make them promising targets for diffusion kernels as measures of affinity between amino acids. The versatility of our method allows the implementation of future kernels with higher performance. The source code of the proposed method is accessible at https://github.com/taherimo/kludo. Also, the proposed method is available as a web application from https://cbph.ir/tools/kludo.

**Supplementary Information:**

The online version contains supplementary material available at 10.1186/s12859-022-04902-9.

## Background

The first step in understanding protein function, evolution, and three-dimensional organization is typically to partition its structure into more elementary units called domains. Protein domains are compact and recurring units of proteins that are able to fold and function independently. Though it has been almost half a century since the introduction of the concept in 1973 by Wetlaufer [1], to define structural domains is still the subject of much debate. Generally, protein domain can be characterized from three different yet related standpoints [2–4]: (1) thermodynamic stability, folding autonomy, and compactness which reflect structural properties, (2) recurrence of conserved and genetically reused traits that represent evolutionary features, and (3) specific role in molecular mechanisms that corresponds to functional semi-independence. The delineation of these structurally meaningful sub-units facilitates many proteomics investigations, including establishing the evolutionary relationship of the structures [5], protein-protein interactions [6], de novo prediction of protein structure and function [7], and molecular dynamics studies [8, 9], which otherwise would be all challenging tasks in a full-length protein.

Though there exist several methods that attempt to predict domain boundaries from amino acid sequences, experimentally determined 3D structures of proteins provides rich information of atom coordinates that makes it a better starting point for delineation of the chains with readily available spatial structure. Generally, there are three types of approaches for the systematic identification of structural domains from 3D structure [10]: manual, semi-manual, and automated. In manually curated classification databases, structural domains are basically assigned by visual inspection of human experts. SCOP [11] is the most extensive database of this kind, which mainly deals with recurrence properties and evolutionary aspects of structural domains. AUTHORS is another database that refers to a set of manually solved domain assignments collected by Islam et al. [12]. Semi-manual databases are those that primarily employ automated methods for protein decomposition, but for instance, in the case of the CATH database, the inconsistencies between the methods in the first stage are resolved by experts’ supervision [13]. Efforts for proposing algorithmic methods for domain identification started almost immediately after introducing the concept itself [14–17]. However, it was not until the debut of Parser for Protein Unfolding Unit (PUU) [18] in 1994 that the use of separate extensive datasets for parameter optimization and evaluation of automated methods became practicable. This, along with DETECTIVE [19], DOMAK [20], and DAD [12] marked the start of the second generation of automated methods for protein domain assignment and were implemented in the CATH database.

Exponential growth in the number of solved protein structures in the last two decades overwhelms human expert inspection and thus favors fully automated approaches [21]. In fact, new algorithms are being introduced almost every year since the first methods were launched. This diverse range of strategies includes but is not limited to graph theoretical approaches [22–27], Gaussian network models [28, 29], Van der Waals interactions and hydrogen bonds analysis [30, 31], Ising models [29, 32], fuzzy clustering [33], and inspection of secondary structures [34–36]. Many methods, however, try to minimize the inter-domain interface and make use of structurally compact regions [37–40]. Veretnik et al. provided a comprehensive comparison of the proposed algorithms [10], and dConsensus implemented a consensus of publicly open methods until 2010 [41].

Despite the rich history of domain assignment techniques, agreement among the different algorithms over a dataset rarely exceeds 80 percent of the consisting structures [42] as it is also the case for unanimity between the expert methods of SCOP and CATH. This mirrors the subjectivity of the task that stems from distinct criteria for domain definition, as described above. SWORD [43] is one of the latest automated methods that addressed this problem by offering alternative decompositions for a protein chain, though producing multiple parsing is not unprecedented among the previous methods [44, 45]. Nevertheless, presenting new algorithmic methods of protein decomposition is desirable since each approach bears certain disadvantages [46] and complex structures are often required to be tackled with different strategies for a convenient partitioning.

From the viewpoint of computer science, protein domain assignment is a clustering problem, therefore as a matter of course, the main focus of the previous methods was the clustering algorithms they employed for protein partitioning. While most authors have tried to develop complex clustering algorithms, but designing a measure of affinity (proximity) between the amino acids that eases the clustering problem has remained rather uncharted.

### Introduction to kernels

Given a set of $n$ data points $\Omega =\{ x_1,...,x_n \}$, a kernel function $k:\Omega \times \Omega \rightarrow {\mathbb {R}}$ expresses affinity between each pair of points in $\Omega$. For any kernel function, there exists an implicit function $\phi :\Omega \rightarrow \mathfrak {H}_k$ that maps every data point $x_i\in \Omega$ to a very high (or possibly infinite) dimensional Hilbert space $\mathfrak {H}_k$ where for each couple of points $x_i$ and $x_j$ in $\Omega$ the kernel function *k* appears as the inner product $k(x_i,x_j )=<\phi (x_i),\phi (x_j)>$. Notice that using the kernel function *k*(., .) we are able to obtain affinity between each pair of points in the unknown space $\mathfrak {H}_k$ without explicitly knowing this space [47]. Heretofore, several kernel functions have been introduced for clustering and classification of data points in various disciplines [48, 49].

Graph node kernels express affinity between each pair of nodes in a graph. A kernel matrix *K* on a graph is a symmetric positive semi-definite matrix, with entries $[K]_{ij}$ as an indicative similarity coefficient between the nodes $v_i$ and $v_j$. Among the graph node kernels already presented, diffusion kernels are most frequently used in the literature [50]. The core idea is to let an initial quantity, like heat, to diffuse from each node to the neighboring vertices. The amount of heat exchanged between the nodes $v_i$ and $v_j$ over a time interval can then be a measure of similarity between $v_i$ and $v_j$. Alternatively, this measure can be perceived as the probability of an initialized random walker on one of the starting nodes to meet another vertex. This essentially allows the measure to be more robust to noises by capturing the affinity through all the connecting paths between the two nodes. Though the primary affinity coefficient is defined locally, diffusion models reveal the graph’s overall structure at greater scales by running the process forward in time. This enables these methods to characterize a sound notion of global similarity besides describing clusters as regions with a low probability of escaping for the random walker.

### Protein decomposition using graph node kernels

Structural domains of proteins are so entangled that, in most cases, clustering of the amino acid residues using the Euclidean distance measure would not lead to appropriate results [43]. In practice, kernel-based clustering methods are very useful when the structure of the individual clusters is highly non-convex or, broadly speaking, when the measures of dispersion or centrality are not valid descriptors of the actual clusters.

If there exists a kernel function that in its reproducing kernel Hilbert space (RKHS), structural domains of proteins become well separated, then protein structures can be parsed into domains without the need to use a complex clustering algorithm. Inspired by this idea, we developed a protein domain decomposition method based on diffusion kernels on protein graphs. We examined all combinations of four graph node kernels and two clustering algorithms to investigate their ability to decompose protein structures into structural domains.

The performance of our method is tested on five of the most widely used benchmark datasets plus a set of protein chains with less than 40% homology based on SCOPe v2.07 [51]. The results are evaluated by a criterion commonly used to evaluate domain assignment algorithms as well as an extrinsic clustering validity measure. Next, based on the evaluations, one of the kernels was selected to contrast KluDo’s accuracy against the four well-known available methods: DomainParser [22, 23], PDP [40], DDomain [39], and SWORD [43]. Moreover, we have discussed how the power of our method to provide alternative partitioning for a protein structure can address the concept of uncertainty in protein delineation and boost its compatibility with the various interpretations of a structural domain.

## Methods

**Fig. 1:**
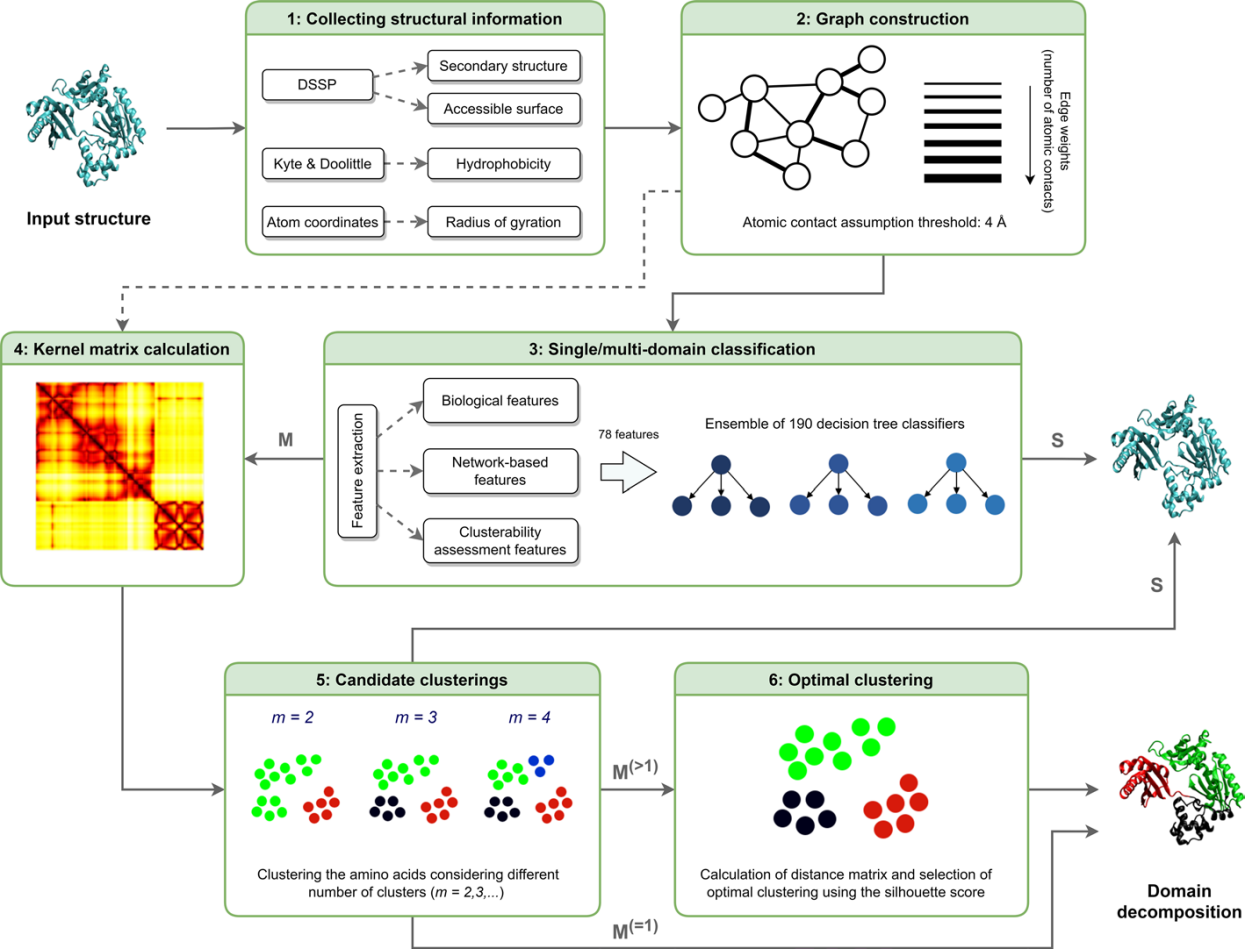
The overall workflow of KluDo. Each box represents a step in the KluDo pipeline. The arrows link algorithmically consecutive steps. **S** and **M** stand for single- and multi-domain, respectively. Also, **M**$^{(={\textbf {1}})}$ and **M**$^{(>{\textbf {1}})}$ show a single clustering and a set of candidate clusterings, respectively. The arrow from the step 5 (candidate clusterings) to single-domain output is drawn to show the cases for which no possible clustering exist and thus the protein structure is reconsidered as single-domain. The dashed arrow from the step 2 to the step 4 shows a special case where the number of domains is specified beforehand, so there is no need to use the single/multi-domain classifier

In this section we present our proposed method: Diffusion Kernel-based Graph Node Clustering for Protein Domain Assignment (KluDo). For a protein chain this method consists of 6 steps: (1) collecting structural information, (2) graph construction, (3) single/multi-domain classification, (4) kernel matrix calculation, (5) obtaining candidate clusterings, and (6) determining the number of domains. Figure 1 illustrates the overall workflow of KluDo for protein domain partitioning.

### Collecting structural information

In the first step, required structural information about the protein structure are obtained. Secondary structure and accessible surface area information are extracted from the protein structure using the DSSP tool [52, 53]. Relative accessible surface for each residue is calculated via dividing its accessibility value to its maximum possible accessibility provided by Miller et al. [54]. The hydrophobicity of each residue is also assigned according to the Kyte-Doolittle scale [55]. Finally, radius of gyration [56] of the protein structure is calculated.

### Graph construction

A weighted undirected graph is constructed based on the protein structure such that each node represents an amino acid residue, and each pair of nodes is connected through an edge if there exists at least one atomic contact between the two amino acids. In our case, it is assumed that two atoms are in contact if their Euclidean distance is equal to or less than 4 Å. The number of atomic contacts between each pair of residues is considered as the weight of the edge between the two corresponding nodes. Further, to evaluate the plausibility of our approach for constructing the protein graphs a set of randomization tests were performed (see Additional file 1).

### Single/multi-domain classification

In this step, a bagging (bootstrap aggregating) classifier is used to categorize the input protein structure into either single or multi-domain classes. The classifier consists of a set of weak binary classifiers each trained on a balanced bootstrap sample of the training set (described below). This way, balanced sets are generated (by resampling) while avoiding to neglect any part of data (by multiple bootstrapping).

To provide training data for the classifier, we subtracted ASTRAL40 (v2.07) and five well-known protein domain assignment benchmark datasets (described in the section “Test datasets”) from ASTRAL95 (v2.07), which led to a set of 13,350 proteins. From these proteins the ones that were considered as single-domain in both SCOP (SCOPe v2.07) and CATH (v4.2.0) were labeled as single-domain and the ones that were considered as multi-domain in either SCOP or CATH were labeled as multi-domain. This resulted in a set of 11,546 protein chains consisting of 6862 single-domain and 4684 multi-domain structures. To overcome the imbalancedness of the two classes a resampling procedure was performed on each bootstrap before training (see Additional files 1, 2).

Based on an 80%-20% train-test split evaluation, decision tree was chosen as the base estimator type from a set of candidate models. Also, the bootstrap size was set equal to the size of the training set to cover all data as much as possible. In order to set the two other hyper-parameters, namely, the number of decision trees and the resampling procedure (to balance bootstrap samples), a grid search over the training set using 5-fold cross-validation was performed. As a result, the combination of 190 decision trees and SMOTE resampling algorithm [57, 58] was selected as the best choice (see Additional file 1).

**Fig. 2:**
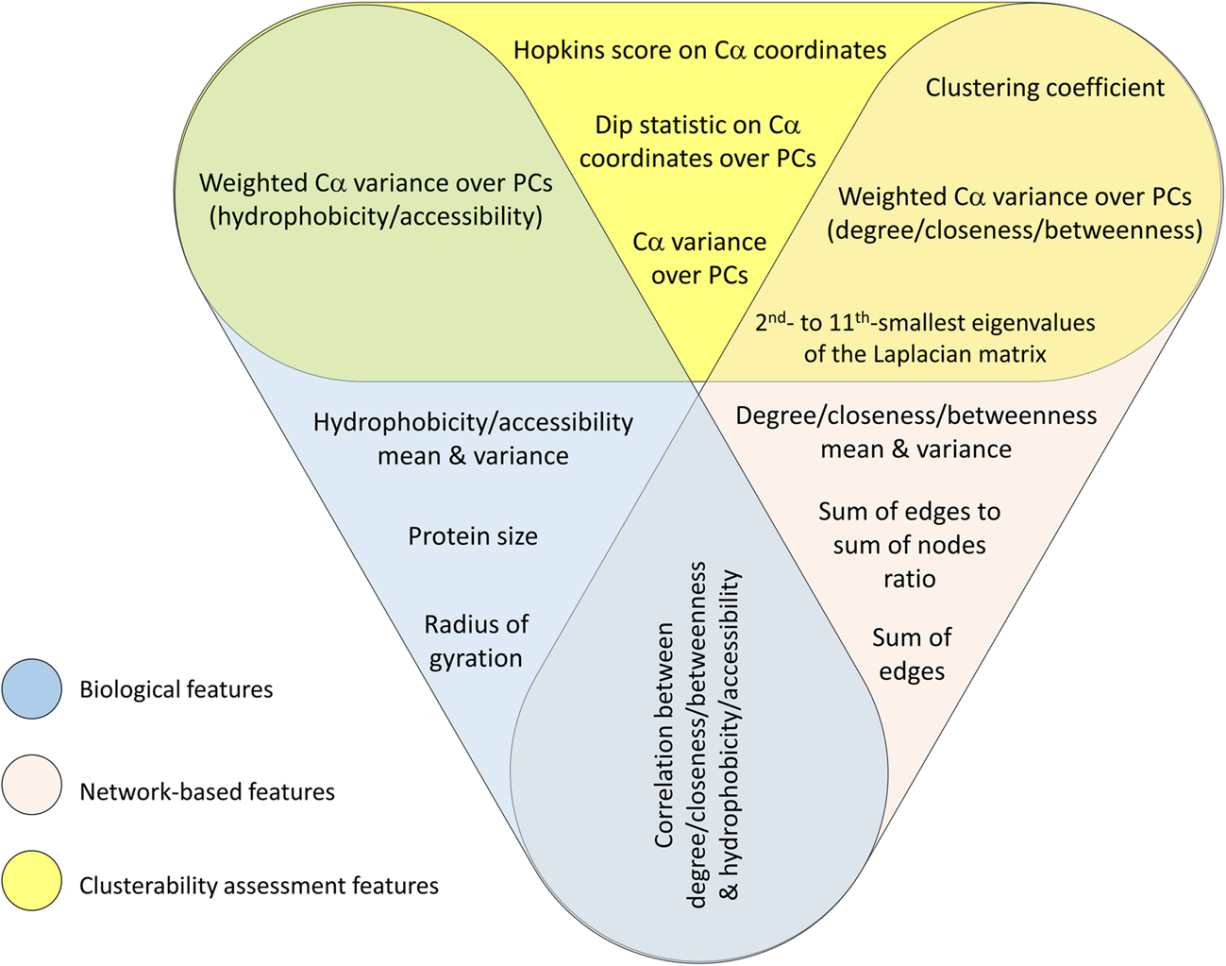
Venn diagram of the input features for the single/multi-domain classifier. Each label represents a group of one or more features (refer to Additional file 1)

The input features for the single/multi-domain classifier can be grouped into three (overlapping) types: (1) biological features, (2) network-based features, and (3) clusterability assessment features. Figure 2 shows a Venn diagram of the feature sets. In general 78 features are used for the single/multi-domain classifier (see Additional file 1). According to Fig. 2, a portion of the features are quite self explanatory: the protein size (in terms of the number of residues), the radius of gyration [56] (as a measure of protein compactness), mean and variance of hydrophobicity and surface accessibility of the residues, sum of edges and the ratio of the sum of edges to the number of nodes.

To calculate 42 of the features, three network centrality measures are used: degree, closeness [59] and betweenness [60]. Also, to calculate 30 of the features (most of which involve variance of alpha-carbon coordinates as a measure of clusterability), a principal component analysis (PCA) is performed on alpha-carbon coordinates. Moreover, Hopkins [61] and dip statistics [62] (as the measures of clusterability) are computed based on alpha-carbon coordinates. In addition, network clustering coefficient (global and average local) [63, 64] is calculated as a measure of network clusterability (see Additional file 1).

Since the constructed network in the previous step is weighted, in most cases there are two weighted and unweighted versions for the network-based features. Also as a variance can be calculated in two weighted and unweighted manners, a set of biological and network-based values are considered as weights when calculating weighted variances (see Additional file 1).

### Kernel matrix calculation

In this step, a kernel matrix is calculated according to the one of the graph node kernels described below. In this study four graph node kernels were examined: Laplacian exponential diffusion kernel, Markov diffusion kernel, Markov exponential diffusion kernel and regularized Laplacian Kernel [49].

*Laplacian exponential diffusion Kernel (LED)* is computed as:

1 $$\begin{aligned} K_{LED}= e^{-\beta L} \end{aligned}$$

where $\beta$ (the bandwidth parameter) can both serve as time parameter and scale parameter and *L* is the Laplacian matrix of the network. The value of the parameter $\beta$ is specified according to the section “Bandwidth determination”). The Laplacian matrix *L* is defined as $L=D-A$, where *D* and *A* are degree and adjacency matrices, respectively. In the case of a weighted network, $A_{ij}$ is equal to weight of the edge between the nodes $v_i$ and $v_j$, and zero if the two nodes are not connected. Also *D* is a diagonal matrix with the degree of the vertex *i* for $D_{ii}$ and zero for all off-diagonal elements. For a weighted network, $D_{ii}$ is computed as the sum of the weights of all edges linked to the node *i*. The exponential for the matrix $-\beta L$ can be calculated by the Maclaurin series:

2 $$\begin{aligned} K_{LED}=\sum _{p=0}^\infty \frac{1}{p!}(-\beta L)^p \end{aligned}$$

In fact the term $(-\beta L)^p$ counts the number of paths of length *p* between each pair of nodes while the denominator *p*! normalizes path counts by path lengths. With *L* as a symmetric matrix, $K_{LED}$ becomes a positive semi-definite matrix, as proved in [50].

*Markov diffusion kernel (MD)* defines a discrete-time counterpart of diffusion distance between the nodes of a graph in a diffusion model [65, 66]. With the help of periodic Markov chains, the kernel measures the similarity between the pattern of heat diffusion between a pair of nodes. Thus, a zero distance is assigned when two nodes diffuse through the graph in exactly the same way [48]. MD kernel matrix is computed as:

3 $$\begin{aligned} K_{MD}=Z(t)Z^T(t) \end{aligned}$$

where $Z(t)=\frac{1}{t}\sum _{\tau =1} ^t P^\tau$ with *t* as time (bandwidth parameter) and *P* as transition-probability matrix for the Markov chain process that is defined as $P= D^{-1}A$.

*Markov exponential diffusion kernel (MED)* was introduced to balance the similarity measure in LED with respect to degree of vertices [67]. It modifies LED to prevent assigning higher similarity between central nodes compared to peripheral nodes:

4 $$\begin{aligned} K_{MED}= e^{-\beta M} \end{aligned}$$

where Markov matrix *M* is defined as $M=\frac{D-A-nI}{n}$ with *I* as identity matrix and *n* as the number of vertices in the graph. Here, *n* is in fact the maximum possible degree (i.e. $n-1$) plus one for a simple (unweighted) graph. Since in the proposed method a weighted graph is constructed, naturally we need to substitute *n* with the potential maximum weighted degree plus one. Nevertheless, in this study, we considered de facto maximum weighted degree plus one instead of *n*.

*Regularized Laplacian kernel (RL)* was first designated in the context of regularization operators as a kernel that counts all the paths between a couple of nodes in a graph, regardless of the path length [68] (refer to Eq. 2). This similarity measure can also be interpreted as relative forest accessibilities between nodes in terms of matrix-forest theorem [69]:

5 $$\begin{aligned} K_{RL}=\sum _{p=1}^\infty (-\beta {L})^p=(I+\alpha L)^{-1} \end{aligned}$$

where $0\le \beta \le 1$ (and equivalently $\alpha >0$) limits the number of the edges in each forest as described in [69]. This kernel is also closely related to the well-known random walk with restart similarity and the commute-time kernel [70].

#### Bandwidth determination

Each of the four kernel functions possess a bandwidth parameter that its magnitude can be interpreted as the time of the diffusion or its corresponding random walk. Thus, this pre-set time must accord with the size of the graph so that the random walker has enough time to search the whole protein. Since our primary criterion for laying the edges in the graph is the Euclidean distance between each pair of residues (atomic contact), the radius of gyration [56] of a protein structure can be a proper indicator for the size of its respective graph. For a protein, this value is defined as the root mean square distance between each atom of the structure to its centroid and is proportional to the number of residues to the power of 0.5 to 0.6 [71]. The displacement length of a random walker on the graph, on the other hand, is proportional to the square root of the time of the random walk. Therefore, it is a reasonable approximation to assume a simple quadratic relationship between the bandwidth parameter and the radius of gyration, i.e. $\eta \times{R_g}^2$ where $R_g$ is the radius of gyration and $\eta$ is the proportionality constant. For each combination of a kernel function and a clustering method, to choose the value of $\eta$, the accuracies resulted of using predefined sets of values for $\eta$ were calculated over the multi-domain structures (assigned as multi-domain by both SCOP and CATH) of the training set provided in the section “Single/multi-domain classification”, assuming all structures as multi-domain. For each kernel function and clustering method composition, three values of $\eta$ that led to highest accuracies were obtained, and among them, the value that best performed over the multi-domain structures of ASTRAL40 was selected. The determined values of $\eta$ as well as the other parameter values of KluDo can be found in Table 1 (see Additional file 1).

**Table 1:** Default parameter values

Parameter	Default value	Description
${\eta }_{{LED}}$	$6 \times 10^{-3}$ (KK), $4 \times 10^{-3}$ (SP)	The bandwidth coefficient of the LED kernel
${\eta }_{{MD}}$	$2.5 \times 10^{-1}$ (KK), $8 \times 10^{-1}$ (SP)	The bandwidth coefficient of the MD kernel
${\eta }_{{MED}}$	$4 \times 10^{-1}$ (KK), $3.5 \times 10^{-1}$ (SP)	The bandwidth coefficient of the MED kernel
${\eta }_{{RL}}$	$2.1 \times 10^{-2}$ (KK), $2.2 \times 10^{-2}$ (SP)	The bandwidth coefficient of the RL kernel
*MDS*	27	The minimum domain size
*MHS*	30	The maximum alpha-helix size to merge
*MSS*	27	The minimum segment size
*SDR*	1.5	The maximum segment to domain count ratio

For each kernel function a couple of values are provided for $\eta$ (the proportionality constant of the bandwidth parameters) that indicate the values for kernel k-means (KK) and spectral clustering (SP), respectively

### Obtaining candidate clusterings

Given a kernel matrix obtained in the previous step, several clustering algorithms (desirably kernel-based) can be used to parse the protein structures. In this study we incorporated two clustering algorithms: kernel k-means [72] and spectral clustering [73]. Both of these algorithms take the number of clusters, *m*, as an input parameter. In our method, starting from $m=2$, clustering is performed for larger values of *m* to the extent that is feasible (considering the the parameter values).

The kernel k-means algorithm works like the regular k-means algorithm in the feature space corresponding to a kernel function. Since cluster centers are not obtainable from the kernel matrix, the kernel k-mean algorithm uses a kernel trick to implicitly calculate the distance of the points to cluster centers. To reduce the sensitivity to random initial partitioning, we repeat the algorithm for 100 times and then select the best output in terms of within-cluster sum-of-squares.

Also, spectral clustering operates on a similarity matrix (in our case, kernel matrix). It first forms a similarity graph based on the similarity matrix, then uses spectral decomposition of the graph Laplacian to obtain a spectral embedding of the points. Finally it employs a clustering algorithm to cluster the points based on their spectral embedding. For this purpose we chose the standard k-means algorithm. Similar to using the kernel k-means algorithm, here the k-means algorithm is also repeated for 100 times and the best partitioning is selected.

**Fig. 3:**
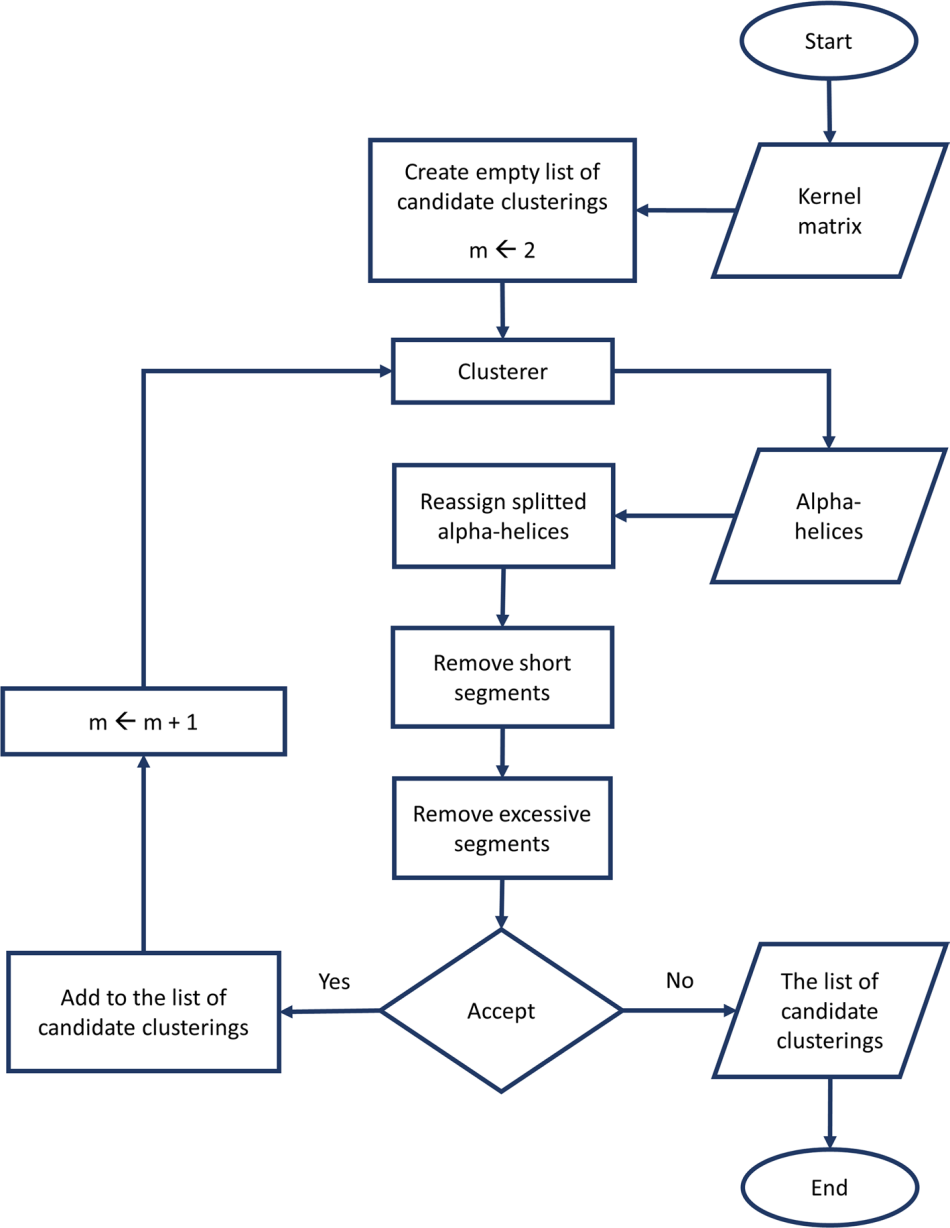
The flowchart of obtaining candidate clusterings. The figure depicts the procedure of obtaining the set of candidate clusterings. Based on the kernel matrix (calculated in the previous step), for each value of *m*, the protein structure is first split into *m* clusters (using either kernel k-means or spectral clustering). Next, the divided alpha-helices with a maximum size of *MHS* are merged with their adjacent segments. In the subsequent steps the short and excessive segments (with respect to the *MSS* and *SDR*, respectively) are removed. The process is then repeated with increasing *m* until the generated partitioning is rejected according to the *MDS* parameter (designated as the accept step in the flowchart)

Due to nature of the protein structures, some post-processing procedures are necessary after clustering of the amino acids. Figure 3 shows the flowchart of the current step including the post-processing procedures. A clustering of a protein chain of size *n* can be shown by a list of cluster labels: $L=<l_1,l_2,\ldots ,l_n>$ where $l_i$ shows the cluster label of *i*th residue in the protein chain. Following each clustering, to avoid splitting alpha-helices among multiple domains, each divided alpha-helix of size less than or equal to the parameter *maximum alpha-Helix size to merge* (*MHS*) is reassigned to the domain that comprises the major fraction of its residues (*L* is updated).

Given the list *L*, the protein chain can be sliced into segments such that each segment consists of a set of consecutive residues that belong to the same cluster and no two consecutive segments are members of the same cluster. If we denote the number of segments by *t* it is obvious that $t \ge m$. The segments can be shown by an ordered list $S=<s_1,s_2,...,s_t>$ in which $s_i=(b_i,e_i)$ where $b_i$ and $e_i$ represent index of the first and last residues of the segment in the protein sequence, respectively.

*Minimum segment size (MSS)* is a main parameter of our algorithm that implies there should not be any segment with the size less than this value. As a result of clustering, short segments (with the size less than *MSS*) may be generated, which are removed in a greedy manner: starting with the shortest, the segment is merged with the adjacent ones. The procedure is repeated until no short segment is left. The *maximum segment count to domain count ratio* (*SDR*) serves as another input parameter of our algorithm. To meet this condition, the process of removing short segments is continued until the given ratio is fulfilled (designated as remove excessive segments in Fig. 3). Algorithm 1 describes the procedure of removing short segments.
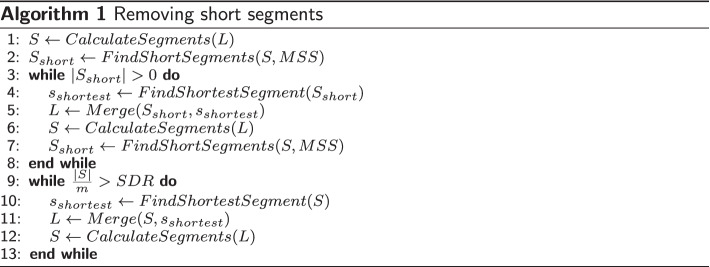


In Algorithm 1 the Merge function takes a list of segments *S* and a segment $s_i$ as input and merges the segment $s_i$ with its adjacent segments ($s_{i-1}$ and/or $s_{i+1}$) according to the procedure that is shown in Algorithm 2. The function ${\bar{d}}(.,.)$ denotes the distance between a pair of segments, which in this study is the average distance between all pairs of residues in two segments. In other words, if we denote the matrix of distances between all residue pairs by *D*, the distance between two segments is calculated as:

6 $$\begin{aligned} {\bar{d}}(s_k,s_p)=\frac{\sum _i^{s_k}\sum _j^{s_p}[D]_{ij}}{|s_k|.|s_p|} \end{aligned}$$

The distance matrix *D* that shows the distance between all pairs of residues is calculated as:

7 $$\begin{aligned} {[}D]_{ij}=[D]_{ji}=\sqrt{[K]_{ii}-2[K]_{ij}+[K]_{jj}} \end{aligned}$$

where *K* is one of the kernel matrices described in the section “Kernel matrix calculation”. In Algorithm 2, the predecessor (successor) of a segment will be assumed to be NULL if the segment is at the start of (end of) a protein chain. Also the cluster label of a segment $s_i$ is denoted by $L[s_i]$.
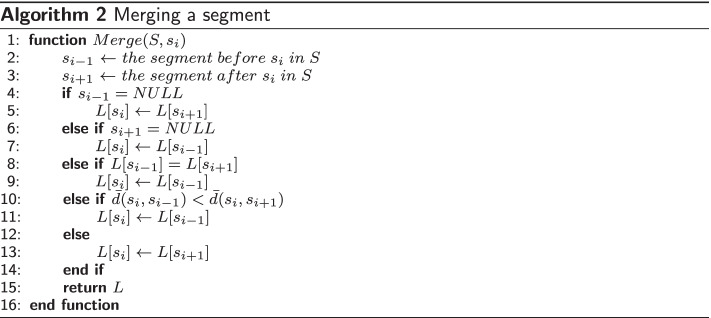


For a clustering that consists of ${m}$ clusters ${C_1,C_2,\ldots ,C_m}$, if there exists a cluster $C_i$ with the size less than the *minimum domain size* (*MDS*) parameter (i.e. $|C_i|<MDS$), the whole clustering is rejected. As a general rule, the clustering process is dismissed if no feasible partitioning exists with respect to the input parameters of our method. In such case all the obtained clusterings up to $m-1$ are considered as candidates. Otherwise the algorithm proceeds with $m+1$. Note that if there is no feasible partitioning for $m=2$, the algorithm rejects the multi-domain assumption of the single/multi-domain classifier and the chain is reconsidered to be single-domain.

### Determining the number of domains

Given a set of candidate clusterings (obtained from the previous step) and a matrix of pairwise distances between the residues (calculated using the Eq. 7) optimal number of domains is calculated in this step. To do so, we only consider hydrophobic amino acids (with a hydrophobicity index greater than 2) to calculate optimal clustering since their distribution is a stronger measure of structural modularity. So, from *n* amino acids, $n_h$ hydrophobic residues are selected ($n_h<n$). Then the silhouette index [74] (a clustering validity measure) is calculated for each candidate clustering as:

8 $$\begin{aligned} SL_m=\frac{1}{n^h}\sum _{i=1}^{n_h}\frac{b(i)-a(i)}{\max {\{a(i),b(i)\}}} \end{aligned}$$

where *a*(*i*) is the mean distance between the *i*th amino acid and all other amino acids in the same cluster and *b*(*i*) is the smallest mean distance of the *i*th amino acid to all amino acids in any other cluster, of which *i*th residue is not a member. Finally, the number of domains, is chosen such that the silhouette score is maximized:

9 $$\begin{aligned} m_{opt}={\mathop {\mathrm{argmax}}\limits _m}\{SL_m\} \end{aligned}$$

## Results and discussion

### Assessment method

In this study two methods were used to measure the correctness of a predicted decomposition with respect to a target one: domain overlapping score and adjusted Rand index. For both of these measures (described below) a certain threshold can be used to consider a predicted assignment as true. We used SCOP (SCOPe) [51] and CATH [75] assignments as the references for the evaluation. More precisely, we considered an assignment as true if it accords with the assignments in SCOP or CATH databases.

#### Domain overlapping score

**Fig. 4:**
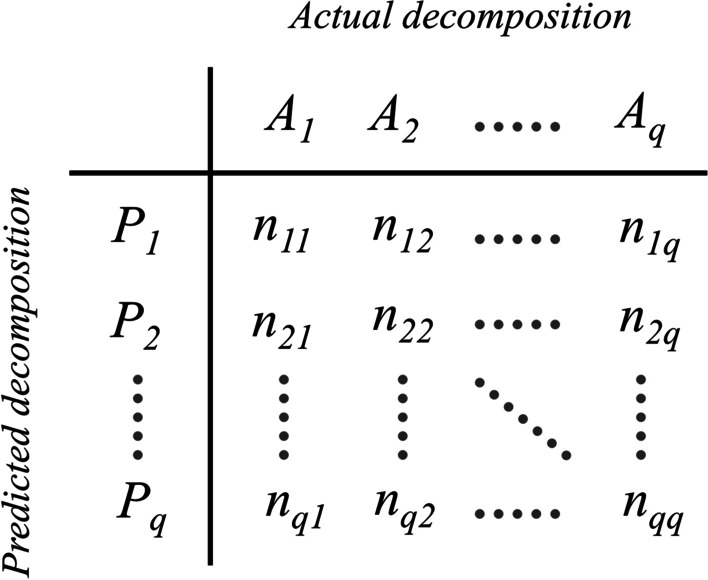
Overlap table. For a pair of domain decompositions (predicted versus actual) with equal number of domains (*q* domains), an overlap table is a square $q\times {q}$ matrix in which each element $n_{ij}$ shows the number of the amino acids that are members of the domain *i* in the predicted decomposition and the domain *j* in the actual decomposition

This method is first presented by Jones et al. [76]. To compute the overlapping score (OL), a one-to-one optimal matching between the identified and target domains is first established (using the overlap table [76]). Then the percentage of the residues that fall in the same domains in both assignments is considered as the OL score. In other words, given a pair of predicted and true domain decompositions (depicted by *A* and *P* in Fig. 4, respectively) for a protein of size *n*, one can form an overlap table in which each element $n_{ij}$ shows the number of the residues that are members of the domains *i* and *j* in the predicted and true decompositions, respectively. After obtaining the optimal matching between the two decompositions, OL score is calculated as:

10 $$\begin{aligned} OL=\frac{\sum _{ij\in {M_{opt}}}{n_{ij}}}{n} \end{aligned}$$

where $M_{opt}$ is the optimal matching between the two assignments. An optimal matching is a matching *M* where the overlapping between the two partitionings is maximized:

11 $$\begin{aligned} M_{opt} = {\mathop {\mathrm{argmax}}\limits _{M}}{\sum _{(i,j)\in {M}}{n_{ij}}} \end{aligned}$$

Thus, an assignment is considered to be true if: (1) the number of domains complies with the target assignment, and (2) the overlapping between the identified and reference assignments is not less than a certain threshold. In this paper the threshold of 85% is used to report the main results.

#### Adjusted Rand index

**Fig. 5:**
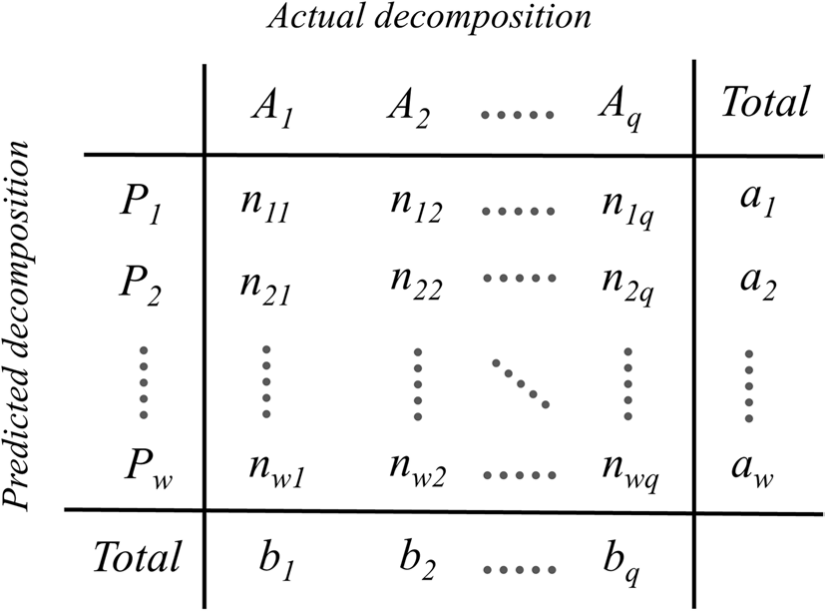
Clustering contingency table. For a pair of domain decompositions (predicted versus actual) with *w* and *q* domains, respectively, a contingency table is a ${(w+1)}\times {(q+1)}$ table (including summation row and column) in which each element $n_{ij}$ shows the number of the amino acids that are members of the domain *i* in the predicted decomposition and the domain *j* in the actual decomposition. Also, $a_i$ and $b_i$ show the sum of the values in *i*th row and column, respectively

By having ground-truth domain decomposition of the protein structures, extrinsic methods of clustering evaluation are applicable to score predicted partitionings. A limitation of the OL score is that it requires equality between the number of domains in the predicted and target assignments whereas there exist several extrinsic clustering validity indices that do not have such a limitation. To evaluate our method, we chose the adjusted Rand index (ARI) [77–79] which is one of the widely used extrinsic metrics to measure clustering performance. Given a protein chain of size *n* and a pair of its domain decompositions (predicted and true decompositions) a contingency table can be calculated in which each element $n_{ij}$ shows the number of the residues that are members of the domains *i* and *j* in the predicted and true decompositions, respectively. So the main diagonal of the contingency matrix shows the pairs of residues that are assigned to the same domains. According to Fig. 5, $a_i$ and $b_i$ are the sum of rows and columns, respectively. ARI was introduced to correct the Rand index (RI) [77] for chance. Based on the contingency table, ARI is calculated as follows:

12 $$\begin{aligned} ARI = \frac{ \sum _{ij} \left( {\begin{array}{c}n_{ij}\\ 2\end{array}}\right) - \left[ \sum _i \left( {\begin{array}{c}a_i\\ 2\end{array}}\right) \sum _j \left( {\begin{array}{c}b_j\\ 2\end{array}}\right) \right] / \left( {\begin{array}{c}n\\ 2\end{array}}\right) }{ \frac{1}{2} \left[ \sum _i \left( {\begin{array}{c}a_i\\ 2\end{array}}\right) + \sum _j \left( {\begin{array}{c}b_j\\ 2\end{array}}\right) \right] - \left[ \sum _i \left( {\begin{array}{c}a_i\\ 2\end{array}}\right) \sum _j \left( {\begin{array}{c}b_j\\ 2\end{array}}\right) \right] / \left( {\begin{array}{c}n\\ 2\end{array}}\right) } \end{aligned}$$

To report the main results in this paper the threshold of 50% is used for the ARI measure. Since it is possible to calculate the ARI measure for any domain decomposition, in this study the performance in terms of mean and standard deviation of the ARI score over each dataset is also presented.

### Test datasets

Five of the most commonly used datasets for protein domain assignments plus a subset of latest release of ASTRAL SCOP were used as test sets. *Benchmark_1* is a set of 467 consensus chains between AUTHORS, CATH, and SCOP provided by Veretnik et al. [80] via excluding chains with more than 90% identity. The chains in *Benchmark_2* and *Benchmark_3* are provided by Holland et al. with some rigorous criteria [2]. The number of domains agrees among SCOP, CATH, and the assignments by authors of the crystallographic or NMR structures in literature. Besides, the included domains were selected as the representatives of different homology groups. The Benchmark_3 further meets the agreement of domain overlap between 3 domain assignments and is more consistent with SCOP and CATH databases. Only half of these two datasets are publicly available by the authors with 156 and 135 chains for Benchmark_2 and Benchmark_3, respectively. The other two sets are the non-redundant set of 90 protein chains with a maximum sequence identity of 30% provided by Islam et al. [12] that here is referred to as *Islam*, and a frequently used benchmark of 55 proteins provided by Jones et al. [76] that here is referred to as *Jones*. Further, we utilized the latest release of ASTRAL SCOPe (version 2.07) to build our most comprehensive non-redundant set by removing the chains with more than 40% sequence identity; here is referred to as *ASTRAL40*. After removing the entries with missing chain IDs, this resulted in a set of 11958 chains, which is also used here to report the performance of different methods based on the number of domains.

### Evaluation of diffusion kernels and clustering methods

**Fig. 6:**
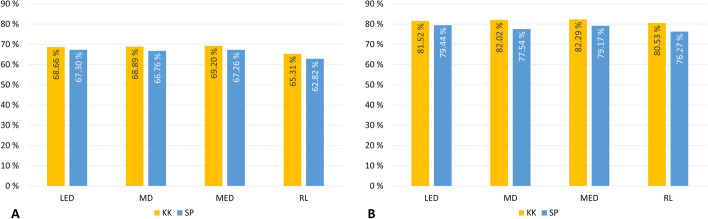
KluDo’s performance in the case of assuming all structures as multi-domain over the multi-domain structures of ASTRAL40. From the ASTRAL40 dataset, 2208 chains that were recognized as multi-domain by both SCOP and CATH were considered. The accuracy (as the percent of true decompositions) for each combination of the four kernels (LED, MD, MED and RL) and two clustering algorithms (kernel k-means and spectral clustering denoted by KK and SP, respectively) is presented. The accuracies are based on the OL and ARI scores with the thresholds of 85% and 50%, respectively (see Additional file 3)

**Fig. 7:**
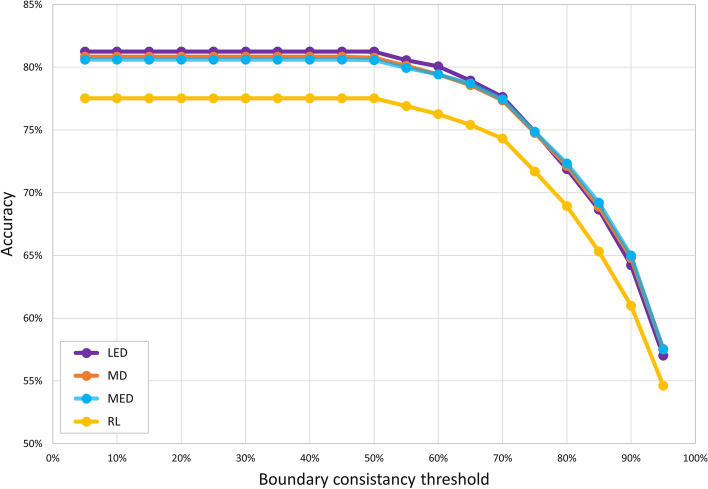
KluDo’s performance in the case of assuming all structures as multi-domain over the multi-domain structures of ASTRAL40 for different thresholds of the OL score. KluDo’s performance for each of the four diffusion kernels with kernel k-means based on a range of thresholds for the OL score (from 5 to 95%) is tested over the set of 2208 structures from ASTRAL40 that were considered as multi-domain by both SCOP and CATH (see Additional file 3)

We examined the capability of KluDo to assign protein domains in the case of using each pair of diffusion kernels (LED, MD, MED and RL) and clustering methods (kernel k-means and spectral clustering) utilizing the parameter values of Table 1. For any of the eight cases, the performance was first measured on multi-domain structures of ASTRAL40 (those considered as multi-domain by both SCOP and CATH) by the assumption that all protein chains consisted of at least two domains. This way, the difference between the efficiency of the kernels/clustering methods is elucidated by eliminating the effect of the single/multi-domain classifier in the overall performance of the method (see the dashed arrow in Fig. 1). Figure 6 shows the accuracies based on the OL and ARI scores (using the thresholds of 85% and 50%, respectively) on 2208 multi-domain chains from the ASTRAL40 dataset. According to the figure, while the kernel k-means algorithm shows a marginally better performance compared to spectral clustering, the three kernels LED, MD and MED exhibit a higher accuracy compared to the RL kernel. Also, no significant difference can be observed in the performance of the three kernels LED, MD and MED. Also, the accuracies based on a range of thresholds of the OL score (from 5% to 95%) in the case of using each of the kernels with the kernel k-means algorithm are presented in Fig. 7. Again, no significant difference in the threshold-independent performance of the LED, MD and MED kernels can be observed while RL under-performs considerably (see Additional file 3).

In order to analyze the performance of the whole pipeline (including the single/multi-domain classifier), KluDo was executed on the five widely used benchmarks plus the ASTRAL40 dataset. According to Table 2, unlike the previous analysis, here spectral clustering has performed better in most cases compared to kernel k-means. Also, in all cases employing the LED kernel has led to a better or equal performance compared to the other three kernels with either of the two clustering algorithms (see Additional files 4–6).

**Table 2:** KluDo’s performance over the test datasets

	LED		MD		MED		RL	
	KK	SP	KK	SP	KK	SP	KK	SP
**Benchmark_1**								
OL	89.9	**91.2**	90.1	90.3	89.7	90.9	88.6	89.7
ARI	92.5	**93.8**	92.5	92.5	92.2	**93.8**	92.0	92.5
**Benchmark_2**								
OL	77.6	**79.5**	76.9	77.6	78.2	**79.5**	74.4	74.4
ARI	85.3	**85.9**	**85.9**	85.3	**85.9**	85.3	84.0	82.1
**Benchmark_3**								
OL	80.7	**83.7**	80.7	82.2	81.5	**83.7**	77.0	79.3
ARI	87.4	**88.9**	87.4	**88.9**	87.4	88.1	85.2	85.2
**Islam**								
OL	88.0	**89.3**	86.7	86.7	**89.3**	88.0	82.7	82.7
ARI	92.0	**93.3**	90.7	**93.3**	90.7	**93.3**	90.7	92.0
**Jones**								
OL	**94.5**	92.7	89.1	92.7	90.9	92.7	90.9	92.7
ARI	96.4	**98.2**	96.4	**98.2**	94.5	**98.2**	**98.2**	**98.2**
**ASTRAL40**								
OL	84.0	**84.7**	84.0	**84.7**	84.1	**84.7**	83.2	83.8
ARI	87.3	**87.8**	87.4	87.1	87.4	87.7	86.9	86.9

KluDo’s accuracy for all combinations of the four kernels (LED, MD, MED and RL) and two clustering algorithms (kernel k-means and spectral clustering denoted by KK and SP, respectively) against the datasets Benchmark_1, Benchmark_2, Benchmark_3, Islam, Jones and ASTRAL40. The accuracies are based on the OL and ARI scores with the thresholds of 85% and 50%, respectively. The maximum accuracy in each row is illustrated in bold

**Fig. 8:**
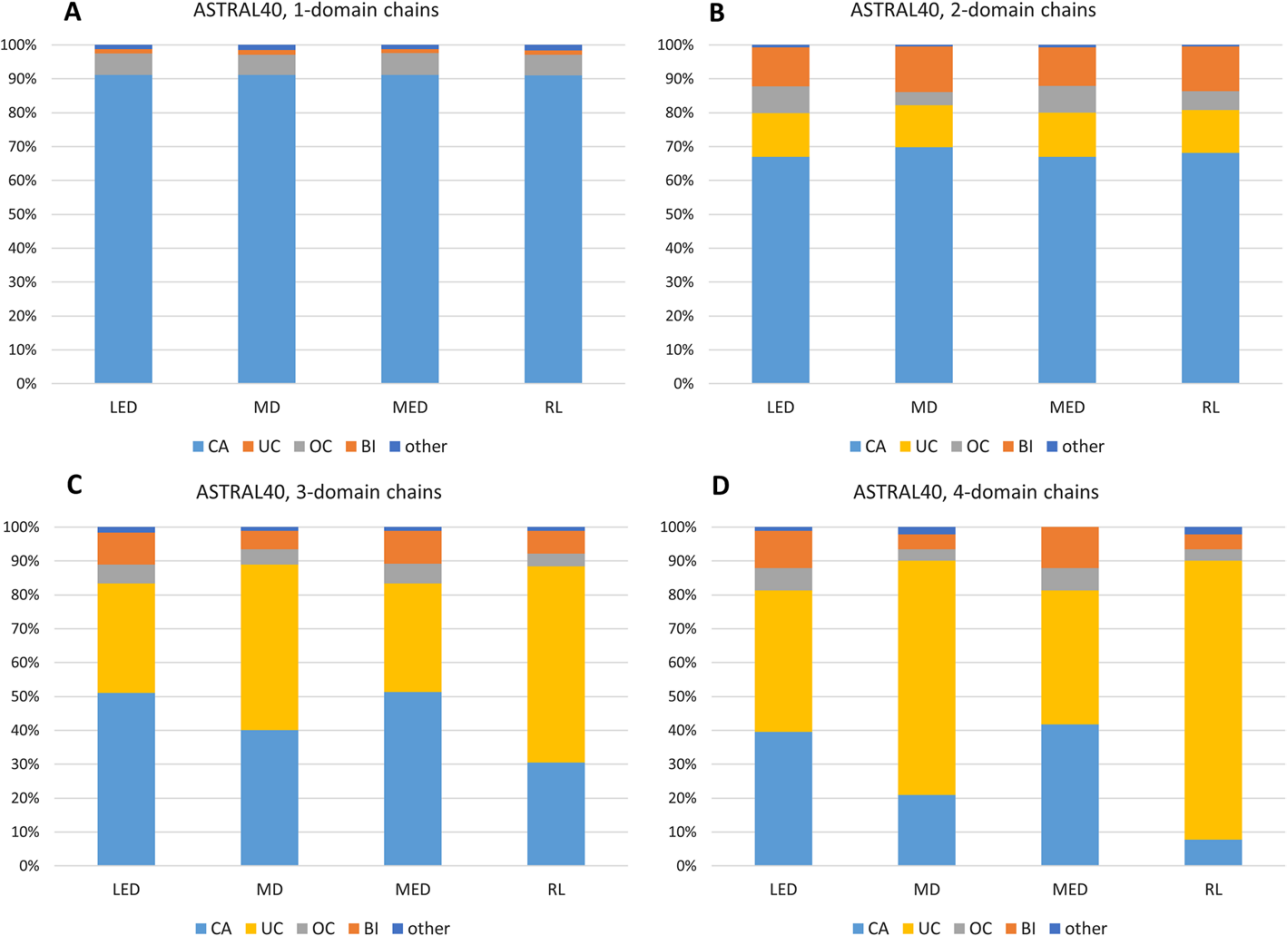
KluDo’s performance based on the number of domains over ASTRAL40. For each of the four diffusion kernels alongside spectral clustering, the plots **A** to **D** show the percent of the correct assignments (the cases of compliance with SCOP or CATH based on the OL score using an 85% threshold), overcuts (the cases of assigning a higher number of domains than both SCOP and CATH), undercuts (the cases of fewer domains than both SCOP and CATH), boundary inconsistencies (the cases of incorrect assignment where the number of domains complies with SCOP or CATH) and other cases, over the 1- to 4-domain subsets (based on SCOP) of ASTRAL40. CA, OC, UC, and BI represent correct assignments, overcuts, undercuts and boundary inconsistencies, respectively (see Additional file 7)

Finally, we inspected the performances over four extracted subsets of ASTRAL40 based on the number of domains. The result of the benchmarking using the spectral clustering algorithm and the OL score (with the threshold of 85%) on each subset (based on the SCOP assignments) is summarized in Fig. 8. Incorrect assignments are sorted into overcuts (assigning a higher number of domains than both of the SCOP and CATH assignments), undercuts (fewer domains than both of the SCOP and CATH assignments), boundary inconstancies (less than 85% domain overlapping with either of the SCOP or CATH assignments) and other cases. All kernels show fairly similar precision on single- and two-domain chains. The majority of erroneous delineations in multi-domain chains consist of undercuts and overcuts which in most cases are related to the performance of the single/multi-domain classifier. Also, boundary inconsistencies which are related to the clustering procedure have a low contribution in the false decompositions of the 3- and 4-domain chains (see Additional file 7).

### Comparison with the other methods

Because of the relative superiority of the LED kernel compared to the other ones in the preceding evaluations, in the next step, we contrasted the performance of KluDo in the case of using the LED kernel (with both of the clustering methods) against four well-known available methods: DomainParser [22, 23], PDP [40], DDomian [39], and SWORD [43]. *DomainParser* works based on recursively bipartitioning of a weighted graph (flow network) using the Ford-Fulkerson algorithm: after setting a pair of artificial source and sink nodes in the protein graph, the minimum set of edges that disconnects the source and sink nodes are removed which results in two sub-graphs. This procedure is then repeated on each sub-graph until its stopping criteria are satisfied. DomainParser tries to avoid splitting of alpha-helices and beta-sheets using its graph construction procedure and stopping criteria. *PDP* attempts to decompose protein structures into smaller fragments based on the assumptions of compactness. By means of a series of cuts, it tries to maximize the inter-domain to intra-domain contact ratio. Another objective of the method is optimizing the number of expected contacts for a domain based on its surface area. In PDP, alpha-helices and beta-sheets are more prone to be split into different domains, compared to DomainParser. Though proper domain boundaries occasionally fall within secondary structures, this feature, besides the compactness assumption, may lead to faulty divisions of proteins in the case of loose structures. Similar to PDP, the *DDomain* algorithm also divides protein structures by maximizing the intra-domain contacts. However, this method uses a pairwise statistical potential based on a normalized contact-based domain-domain interaction profile rather than mere contact count in PDP. DDomain limits each structural domain to a continuous segment, which is not a valid assumption for many protein chains. *SWORD* makes use of evolutionarily preserved substructures (obtained by protein peeling [81]) to reconstruct protein domains in an agglomerative approach. These protein units represent protein architecture at a scale between secondary structure elements and domains. Alternative assemblies of these components allow multiple decompositions for a chain, which is introduced as a measure of ambiguity for a protein structure in this method. Unlike KluDo that uses flat clustering methods, all four described methods are hierarchical methods, from which DomainParser, PDP and DDomain are top-down and SWORD is bottom-up.

**Fig. 9:**
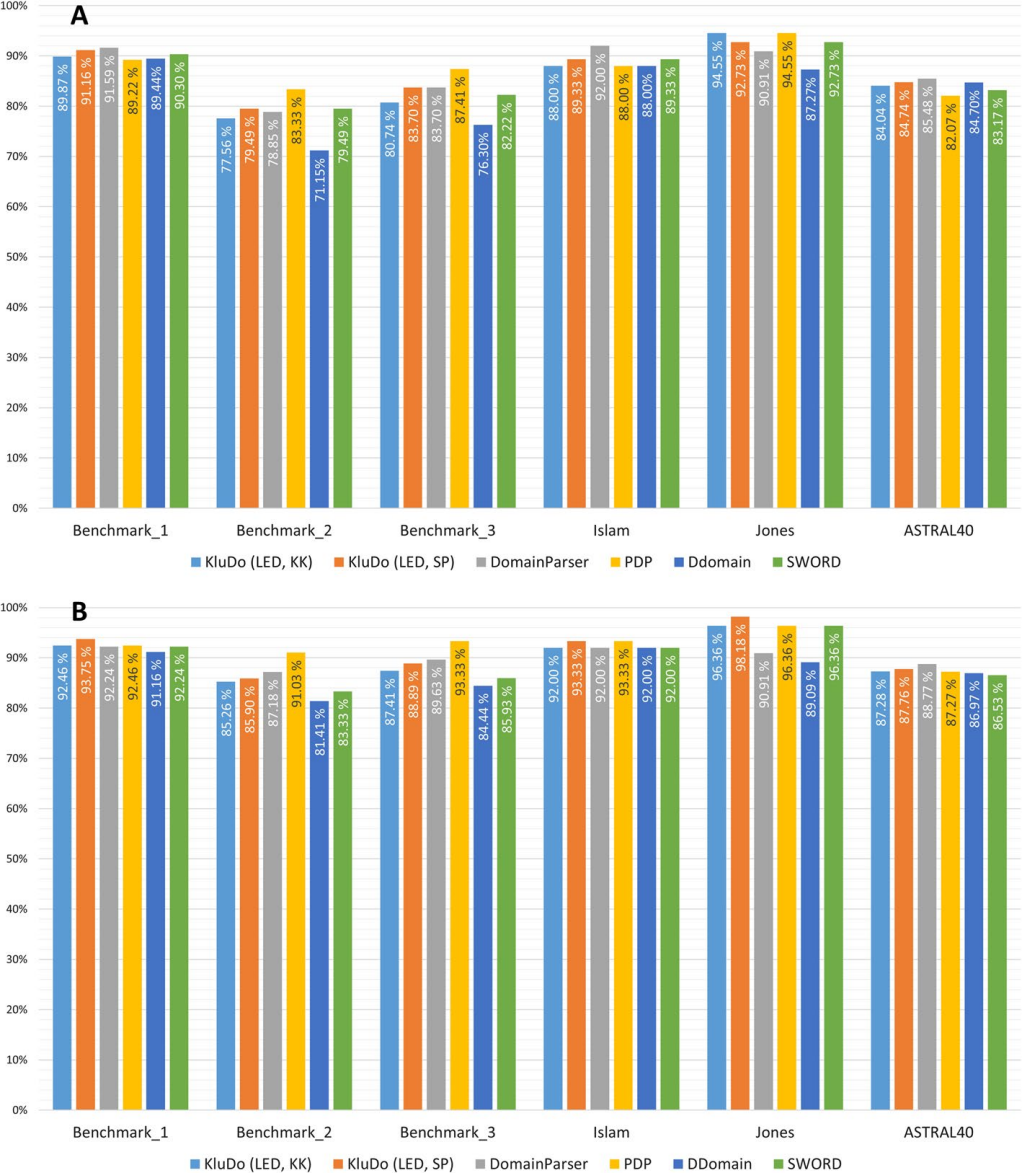
Comparison of KluDo’s performance with that of four automatic methods over the test datasets. KluDo’s accuracy in the case of using the LED kernel with the two clustering methods is compared against the methods PDP, DomainParser, DDomain, and SWORD, over the test datasets. The accuracies are plotted based on the (**A**) OL and (**B**) ARI scores, with the thresholds of 85% and 50%, respectively. KK and SP stand for kernel k-means and spectral clustering, respectively (see Additional file 5)

The performance of each method along with KluDo (using the LED kernel and both clustering methods) based on the OL (85% threshold) and ARI scores (%50 threshold) are illustrated in Fig. 9A, 9B, respectively. According to Fig. 9A, KluDo (using kernel k-means) along with PDP could obtain the best accuracy over Jones. Also, in Benchmark_1, KluDo (using spectral clustering) was able to get a score close to the best performing method, DomainParser. KluDo could also achieve the second highest score over the rest of datasets by employing spectral clustering. Moreover, Fig. 9B shows that KluDo in the case of using spectral clustering could attain the best accuracy over Benchmark_1, Islam and Jones (see Additional files 5, 6).

**Table 3:** Comparison of KluDo’s performance with that of four automatic methods over ASTRAL40 separated by the number of domains

	1-domain		2-domain		3-domain		4-domain		Total
	SCOP	CATH	SCOP	CATH	SCOP	CATH	SCOP	CATH	
**KluDo (LED, KK)**	91.1	92.4	70.3	**78.1**	44.7	48.2	16.5	27.7	84.0
**KluDo (LED, SP)**	91.1	93.2	66.9	76.4	51.1	52.1	39.6	41.1	84.7
**DomainParser**	93.1	94.7	76.4	75.3	**76.8**	56.9	38.9	35.1	**85.5**
**PDP**	85.8	89.3	**78.4**	74.4	76.4	**57.8**	**58.3**	**47.0**	82.1
**DDomain**	**95.0**	**96.2**	72.1	69.1	69.3	50.7	47.2	43.6	84.7
**SWORD**	90.1	93.1	74.9	71.6	73.9	53.1	50.7	35.1	83.2

KluDo’s accuracy in the case of using the LED kernel with the two clustering methods is compared against four automated methods over ASTRAL40 based on the OL score (considering an 85% boundary consistency threshold). The results are presented both overall and separated by the number of domains according to both SCOP and CATH. The maximum accuracy in each column is depicted in bold. KK and SP stand for kernel k-means and spectral clustering, respectively

Furthermore, Table 3 shows the accuracies based on the OL score (85% threshold) over ASTRAL40, separated by the number of domains (based on both SCOP and CATH). According to the table, using kernel k-means has led to a better performance compared to spectral clustering on the 2-domain structures, and the best performance among all methods over the structures considered as 2-domain by CATH. On the other hand, using spectral clustering has resulted in a better accuracy on 3- and 4-domain structures compared to kernel k-means. Despite having the same single/multi-domain classifier, a minor difference in the single-domain accuracy between the two clustering methods can be observed. This drives by the cases in which the multi-domain partitioning by the clustering algorithm cannot pass the assumed condition for a structural domain. These dismissed decompositions revise erroneous classification of the structures as multi-domain. In addition, high accuracy of the method on single-domain structures suggests the potential role of the false negative predictions (considering the single-domain structures as the negative class) by the single/multi-domain classifier in the general performance of the method. As expected, PDP under-performed on single-domain chains as it tends to split non-compact structures illogically. Thus, the main reason of PDP’s lower precision over ASTRAL40 (Fig. 9A) compared to the other methods is the high single- to multi-domain ratio in ASTRAL40. On the contrary, DomainParser displays an inferior accuracy against the other methods on the 4-domain chains due to its inability to cut through secondary structure elements. Nevertheless, it functions best in terms of overall performance based on the OL score and is only bettered by spectral clustering-powered KluDo on ASTRAL40. According to Table 3, there seems a balance in Kludo’s efficiency over the number of domains in the case of using the LED kernel and spectral clustering combination. In general, the results indicate a competitiveness between the prediction power of the proposed method compared to the best available methods (see Additional files 1, 7).

### Diffusion kernels on protein graphs

Graph representation provides a rich context for processing heterogeneous biological data. Although the graph node kernels used in this study are powerful measures of node similarity, they suffer when applied to sparse graphs with a low number of links. Owing to their nature, which is based on the notion of heat diffusion, they are vulnerable to missing links in such graphs [82]. In the case of protein domains, however, corresponding graphs represent dense structures that contain a well-interconnected network of residues. Thus, diffusion kernels are not affected by noise in the form of missing links when implemented in protein decomposition.

Another limitation of such kernels is their time complexity, which scales more than quadratically in the number of nodes. More precisely, matrix exponentiation in the case of the LED and MED kernels has a computational complexity of $O(n^3)$ where *n* is the number nodes in the graph. The MD kernel computes *t* matrix multiplications, each one with a cost of $O(n^{2.373})$ in the fastest algorithm [83]. The matrix inversion in the RL kernel is also computed with a similar complexity to the matrix multiplication, all of which may be a computational burden with large networks. Again, this is not prohibitive when dealing with protein graphs. A figure in the range of 50 to 2000 residues for the majority of the proteins (with a median around 300 [84]) is well below a restrictive threshold for the computational complexity of the graph node kernels.

**Fig. 10:**
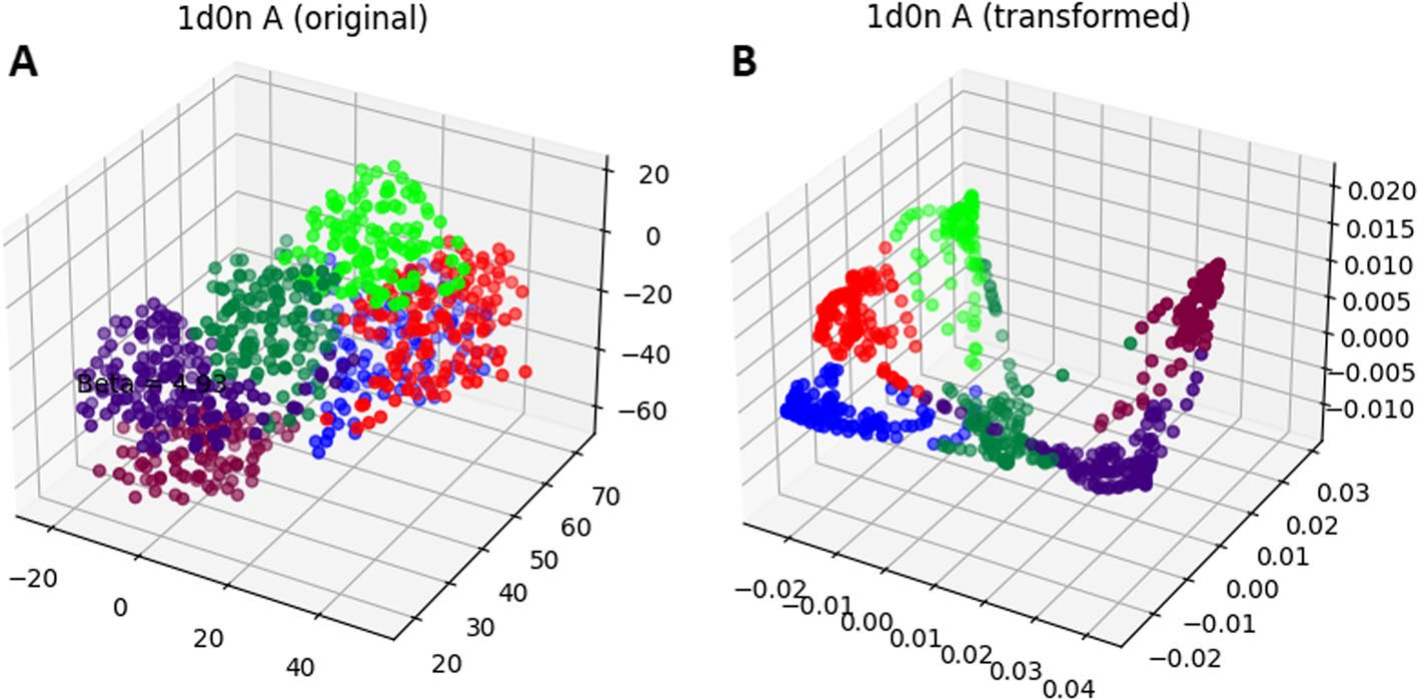
Visualization of a protein structure in RKHS of a kernel. The left plot (**A**) shows 3D structure of a protein (PDB: 1d0n, chain: A) based on center of mass coordinates of its amino acid residues. The right plot (**B**) shows the same structure transformed by the LED kernel (using $0.006\times {{R_g}^2}$ as bandwidth parameter value) and visualized using the three first PCs resulting from kPCA

Also, to intuitively show whether diffusion kernels on protein graphs would lead to a better separation of the structural domains compared to the original 3D structures, the proteins can be visualized in both cases. To view projection of the protein structures in RKHS of the kernels, several methods are applicable from which we use kernel principal component analysis (kPCA) [85, 86]. Figure 10 shows the structure of a protein (PDB: 1d0n, chain: A), in the original 3D space (by considering center of mass of each amino acid as its corresponding data point) and its projection on the first three principal components (PCs) resulting from kPCA over the LED kernel matrix (Eq. 1 with $0.006\times {{R_g}^2}$ as the bandwidth parameter value). The transformed representation shows a relatively better separation of structural domains compared to the original 3D structure.

### Alternative decompositions

**Fig. 11:**
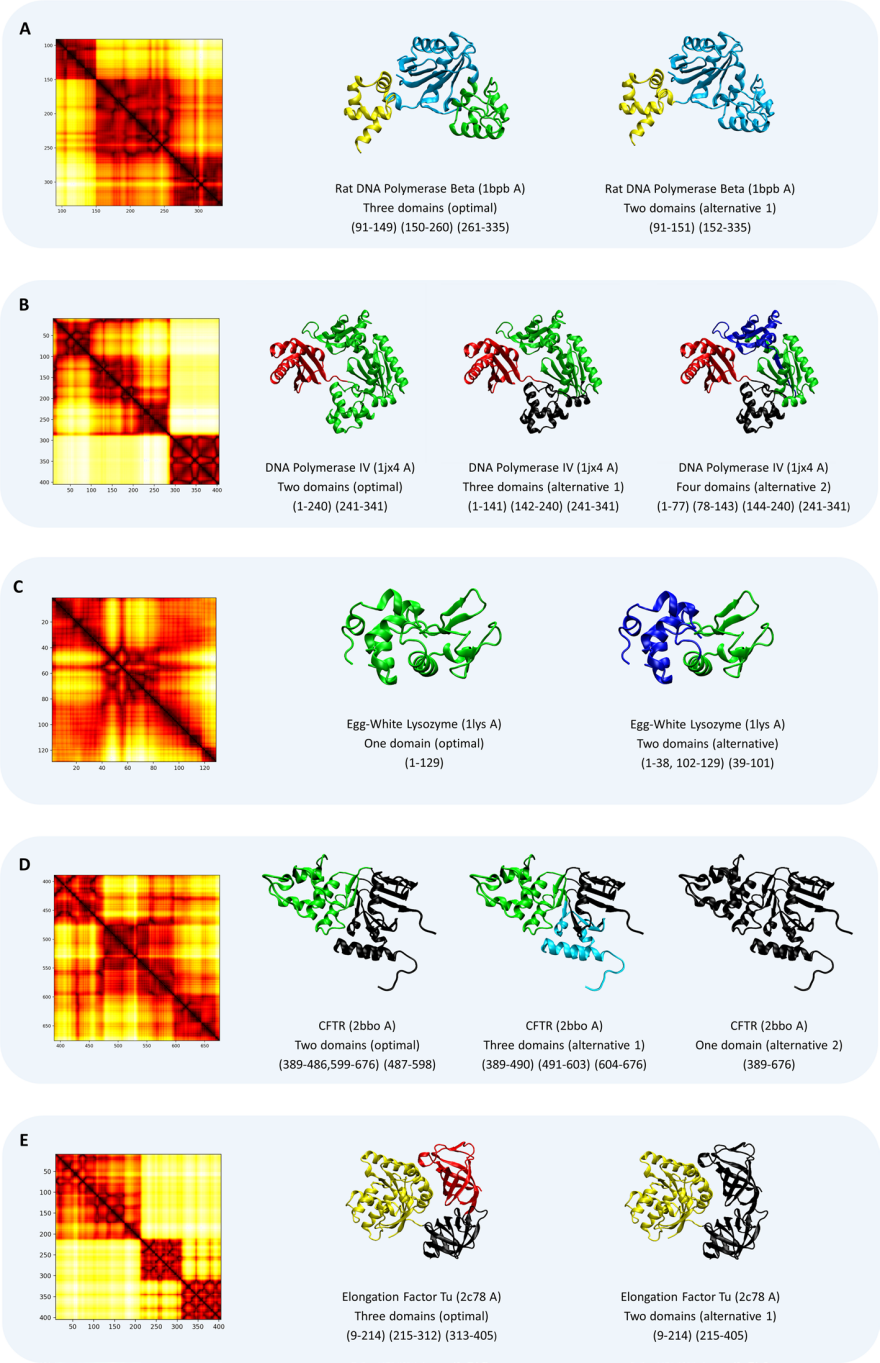
Alternative domain decompositions. **A** to **E** are some practical cases that demonstrate versatility of KluDo in protein delineation. All structures are partitioned using the LED kernel and kernel k-means clustering. The heat maps show the kernel matrices for the protein structures

As described in the “Methods” section, KluDo attempts to delineate the multi-domain protein structures into two up to the highest possible number of domains. The results are then sorted based on the silhouette score over the hydrophobic residues to achieve the optimum partitioning. This generates a number of partitionings that their corresponding silhouette score can serve as a possible index of agreement with experts’ opinion. This enables our method to offer alternative partitioning for the same chain. Multiple decompositions are particularly meaningful in the structures that can be partitioned with different criteria for domain definition or the cases with no consensus parsing among experts. Some examples of such proteins along with their alternative decomposions by KluDo (using the LED kernel and kernel k-means) are presented in Fig. 11.

According to Fig. 11, rat DNA polymerase beta (PDB: 1bpb, chain: A, Fig. 11A) can assumed to be composed of three domains as annotated in SCOP and Sarawa et al. described: fingers (residues 88–151), palm (152–262), and thumb (263–335) [87]. Alternatively, the CATH database partitions this chain into two domains with *palm* and *thumb* parts as a single domain. As it is evident in the heat map, which illustrates the kernel matrix described in the “Methods” section, KluDo can detect both decompositions for this protein. A similar example is DNA polymerase IV (PDB: 1jx4, chain: A, Fig. 11B) with two structural domains in SCOP. Functional interpretation of its crystal structure unveils four domains in accordance with CATH: the finger (1–77), palm (78–166), thumb (167–233), and little finger (244–341) domains occur sequentially from the N to C terminus [88]. Again, KluDo is versatile enough to suggest both alternatives plus a three-domain decomposition all with narrow silhouette score differences. Here the heat map reflects how the first three domains (starting from N terminus) are intertwined with each other.

In the case of egg-white lysozyme (PDB: 1lys, chain: A, Fig. 11C), the structure is annotated as single-domain in both SCOP and CATH. Dynamic simulations and thermodynamics investigations, however, detect two folding units for this chain [89, 90]. KluDo also suggests both the single- and two-domain delineations for lysozyme with a two-segment domain in the latter case. CFTR (PDB: 2bbo, chain: A, Fig. 11D) is another example of capability of KluDo in identifying folding sub-units. Though this chain is annotated as a single-domain structure in both SCOP and CATH databases, fluorescence studies reveal three folding sub-domains for this protein: [91] an N-terminal sub-domain that contains the ATP binding site (389–494), an alpha-helical sub-domain (495–564), and a central alpha/beta core analogous to the F1-type ATPase containing a six-stranded, largely parallel beta-sheet (565–673) [92]. Folding sub-units are of special importance in cystic fibrosis etiology where CFTR folds and misfolds are considered to be the major driver of this genetic disorder. Alternative decompositions may also be helpful in identifying protein motions. In the case of elongation factor Tu (PDB: 2c78, chain: A, Fig. 11E) a three-domain parsing seems to be an obvious choice as is included in both SCOP and CATH. Yet molecular dynamics simulation elucidates how a GTP hydrolysis can induce a large conformational change within the protein [93]. The moving domain is also perceivable by comparing PDB entries for GTP (PDB: 1eft, chain: A) and GDP (PDB: 1tui, homotrimer) bound structures of elongation factor Tu which agrees with alternative partitioning found by our method.

Multi-partitioning enables KluDo to generate a rich library of different decompositions for protein chains. Above examples can certify the value of such library by which the problem of multi-criterial definition of structural domain can be addressed. In contrast to human perception which generally tends to favor only one solution, an automated method that allow more than one way for delimitation of a protein domain can provide multiple avenue of research for complex structures. To the best of our knowledge, only SWORD and DHcL are capable of offering multi-partitioning among automated methods, while the latter one suffers from low accuracy on common benchmarks.

## Conclusions

Protein domain assignment as an ongoing problem for several decades has been tackled by various methods via proposing novel clustering approaches. Alternatively, here we focused on the measure of affinity between amino acids instead of the clustering algorithm. With a competitive accuracy and balanced performance for simple and complex structures (based on Table 3, considering the number of domains as an indicator of protein complexity) despite relying on a relatively naive criterion to choose optimal decomposition, KluDo revealed that diffusion kernels on graphs in particular, and kernel functions in general are promising measures to facilitate parsing proteins into domains and also performing different structural analysis on proteins. Graph node kernels are trending tools widely adopted in real-world applications, particularly in biological data in recent years; examples are gene association studies [67, 94] and PPI network analysis [95]. The size and interconnectedness of protein graphs make them promising targets for diffusion kernels as efficient measures of affinity between amino acids. Besides, developing novel graph node kernels such as the *conjunctive disjunctive* [96] and *MinHash* [83] kernels is currently a hot topic. Further, efforts for refining diffusion kernels may allow attaining higher performance from these techniques. Employing multi-layer graph node kernels [97] and link enrichment [82] are two cases of such studies.

Our proposed approach is a versatile framework that is open to implement more recent graph node kernels (or kernel functions in general) and can offer even greater precision for protein delineation with the help of future progress in this field. Moreover, the capacity of KluDo to propose multiple solutions can tackle the problem of biased study of ambiguous structures that is caused by considering only a single valid domain decomposition for such proteins. The source code of this project (written in Python 3.7) is available on Github at https://github.com/taherimo/kludo. KluDo can be executed as a Windows/Linux/Mac command-line application. We also developed a web application to make KluDo available through the world wide web from https://cbph.ir/tools/kludo. All the parameters in Table 1 besides kernel function, clustering method and lower/upper bound for the number of domains can be set optionally by the users in both command-line and web applications (see Additional file 1).

## Supplementary information

**Additional file 1**. Supplementary document. This document includes additional information about data preparation, the single/multi-domain classifier, bandwidth determination for the kernel functions, randomized graph tests and software tools used for implementation.

**Additional file 2**. Features extracted from the datasets for the single/multi-domain classifier. This file includes the extracted features for the single/multi-domain classifier from the datasets ASTRAL95, ASTRAL40, Benchmark_1, Benchmark_2, Benchmark_3, Islam and Jones. For each protein structure, 78 features were extracted. The last column indicates class label. Refer to Additional file 1for description.

**Additional file 3**. Results and performance evaluation of KluDo over the multi-domain structures of ASTRAL40, assuming all structures as multi-domain. This file contains KluDo results as well as accuracy and mean and standard deviation of the ARI score over the multi-domain structures of ASTRAL40, assuming all structures as multi-domain. The accuracy is calculated based on the OL and ARI scores considering the thresholds from 5% to 100% with the interval 5%. From the ASTRAL40 dataset, 2208 out of 11958 protein chains that were recognized as multi-domain by both SCOP and CATH were considered. All these structures were assumed as multi-domain by KluDo (the single/multi-domain classifier was not employed). The results and performance for all kernel function-clustering method combinations with the default parameter values are provided. KK and SP stand for kernel k-means and spectral clustering, respectively.

**Additional file 4**. Results of KluDo along with the other methods over the test datasets. This file encompasses the results of KluDo along with the other methods (DomainParser, PDP, SWORD and DDomain)over the datasets Benchmark_1, Benchmark_2, Benchmark_3, Islam, Jones and ASTRAL40. KluDo’s results are presented for all combinations of kernel functions and clustering methods using the default parameter values. KK and SP stand for kernel k-means and spectral clustering, respectively.

**Additional file 5**. KluDo's performance along with the other methods in terms of accuracy over the test datasets. This file comprises the accuracies over the results in Additional file 4. Accuracy is calculated based on both of the OL and ARI scores considering the thresholds from 5% to 100% with the interval 5%. In the cases of no agreement in the number of domains between predicted and target assignments, the OL score conventionally was set to -1. KK and SP stand for kernel k-means and spectral clustering, respectively.

**Additional file 6**. Kludo's performance along with the other methods in terms of ARI mean and standard deviation over the test datasets. This file includes mean and standard deviation of the ARI score over the results in Additional file 4 with respect to SCOP, CATH and the mean score based on SCOP and CATH. KK and SP stand for kernel k-means and spectral clustering, respectively.

**Additional file 7**. KluDo's performance based on the number of domains over ASTRAL40. This file consists of the accuracies over the results in Additional file 4 separated by the number of domains. Based on each of the SCOP and CATH databases, four subsets were extracted from ASTRAL40: 1-domain, 2-domain, 3-domain, and 4-domain structures. For each subset, the percent of the correct assignments (the cases of compliance with SCOP or CATH based on the OL score using an 85% threshold), overcuts (the cases of assigning a higher number of domains than both SCOP and CATH), undercuts (the cases of fewer domains than both SCOP and CATH), boundary inconsistencies (the cases of incorrect assignment where the number of domains complies with SCOP or CATH) and other cases were measured. CA, OC, UC, and BI represent correct assignments, overcuts, undercuts and boundary inconsistencies, respectively. Also, KK and SP stand for kernel k-means and spectral clustering, respectively.

## Data Availability

The source code of this project (under MIT licence) is written in Python 3.7 and is available on https://github.com/taherimo/kludo. The web application of this project is also accessible at https://cbph.ir/tools/kludo.

## Abbreviations

ARI: Adjusted Rand index
BI: Boundary inconsistency
CA: Correct assignment
kPCA: Kernel principal component analysis
KK: Kernel k-means
KluDo: Kernel-based clustering for protein domain assignment
LED: Laplacian exponential diffusion kernel
MD: Markov diffusion kernel
MDS: Minimum domain size
MED: Markov exponential diffusion kernel
MHS: Maximum alpha-helix size to merge
MSS: Minimum segment size
OC: Overcut
OL: Overlapping score
PC: Principal component
PCA: Principal component analysis
RKHS: Reproducing kernel Hilbert space
RL: Regularized laplacian kernel
SDR: Maximum segment to domain count ratio
SP: Spectral clustering
UC: Undercut
