# Supplementary material for: Assignment of structural domains in proteins using diffusion kernels on graphs
Source: BMC Bioinformatics. 2022 Sep 8;23:369. doi: 10.1186/s12859-022-04902-9 (PMC9461149; doi:10.1186/s12859-022-04902-9)
Supplement: Supplementary file 1 — Additional file 1. Supplementary document. This document includes additional information about data preparation, the single/multi-domain classifier, bandwidth determination for the kernel functions, randomized graph tests and software tools used for implementation. [file 12859_2022_4902_MOESM1_ESM.pdf]

# Assignment of structural domains in proteins using diffusion kernels on graphs

Supplementary document

## Preprocessing of SCOP assignments

There were very short domains (≤ 10) in the SCOP database (SCOPe 2.07) that appeared to be due to human errors. Hence, before using SCOP assignments for the purpose of training and evaluation, we merged the domains of length 10 residues or less with the adjacent domains using a procedure similar to the one whose pseudo-code is shown in Algorithm 1 (removing short segments). The only difference is that in the cases of different predecessor and successor domains ($10^{th}$ line in Algorithm 2) here we select the shorter domain to merge.

## Training data

In this study we used a set of training data for two purposes: (1) to train the single/multi-domain classifier, and (2) to tune bandwidth parameter of the diffusion kernels. Subtracting ASTRAL40 and five benchmark datasets (Benchmark_1, Benchmark_2, Benchmark_3, Islam and Jones) that were used for evaluation from ASTRAL95 left 13350 protein chains to use for the purpose of training and parameter tuning.

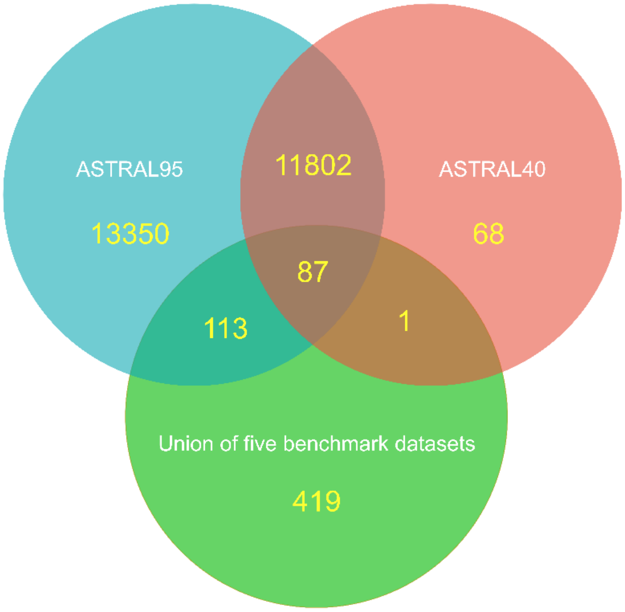


**Figure S1**: Datasets used for training and evaluation.

## Single/multi-domain classifier

An ensemble of classifiers is used to categorize the input protein structure into either “single" or "multi-domain" classes. More precisely it is a bagging (bootstrap aggregating) classifier where each of its estimators (weak classifiers) is trained on a balanced bootstrap sample of the training set. The final output of the classifier is calculated by averaging over the outputs of all decision trees. In this section we describe a detailed explanation about the single/multi-classifier in four sub-sections: feature extraction, hyper-parameter optimization, classifier performance assessment, and feature importance analysis on the classifier.

### Training data labeling

The training set contains the protein chains that there is no an agreement between SCOP and CATH in categorizing them as single- or multi-domain, or their domain decomposition is not available in SCOP and/or CATH. Thus, we used the convention depicted in Table S1 to label such protein chains. The category column assigns a symbol to each case as SS: categorized as single-domain by both SCOP and CATH, MM: categorized as multi-domain by both SCOP and CATH, SM: categorized as single-domain by one of the two databases and multi-domain by the other, S!: categorized as single-domain in one of the two databases and missing in the other, M!: categorized as multi-domain in one of the two databases and missing in the other, !!: missing in both SCOP and CATH. According to the table, we ignored two groups S! and !! to train the single/multi-domain classifier. It led to 6862 protein chains labeled as S (single-domain) and 4684 protein chains labeled as M (multi-domain).

**Table S1:** Training data labeling convention for the single/multi-classifier.

<table>
<tr><th>SCOP</th><th>CATH</th><th>Main label</th><th>Assigned label</th><th>Abundance</th></tr>
<tr><td>single-domain</td><td>single-domain</td><td>SS</td><td>S</td><td>6862</td></tr>
<tr><td>multi-domain</td><td>multi-domain</td><td>MM</td><td>M</td><td>2473</td></tr>
<tr><td>single-domain</td><td>multi-domain</td><td rowspan="2">SM</td><td rowspan="2">M</td><td rowspan="2">1600</td></tr>
<tr><td>multi-domain</td><td>single-domain</td></tr>
<tr><td>single-domain</td><td>no available</td><td rowspan="2">S!</td><td rowspan="2">ignored</td><td rowspan="2">1795</td></tr>
<tr><td>no available</td><td>single-domain</td></tr>
<tr><td>multi-domain</td><td>no available</td><td rowspan="2">M!</td><td rowspan="2">M</td><td rowspan="2">611</td></tr>
<tr><td>no available</td><td>multi-domain</td></tr>
<tr><td>no available</td><td>no available</td><td>!!</td><td>ignored</td><td>9</td></tr>
<tr><td colspan="4">Total abundance</td><td>13350</td></tr>
</table>

### Feature extraction

Here we present a description about each of the features and the way they are calculated. According to Figure 2, three overlapping sets are features are extracted: (1) biological features, (2) The complete list of features along with their description is presented in the Table S1.

*Radius of gyration*

The radius of gyration of a protein structure is an indicator of protein compactness. It is calculated as the root mean square deviation of the atoms' distance from the structure's center of mass. In addition to passing it as an input feature to the single/multi-domain classifier, we use radius of gyration to calculate the bandwidth parameter of the kernel functions.

*Network centrality measures*

To extract 42 out of the 78 features from a protein structure, calculation of three network centrality measures for each node of the protein graph is required: degree, closeness and betweenness. Degree of a node is defined as the number of links incident upon that node. If a network is weighted, then the weighted degree of a node can be computed by summing up the weights of links connected to that node. Closeness [1] of a node is the average length of the shortest paths to all the other nodes in the graph. Betweenness [2] quantifies the number of times a node appears in the shortest path between any pair of nodes. Since the network is weighted, each centrality measure is calculated in two weighted and unweighted (without considering network weighs) cases.

*Weighted variances*

In the cases that we calculate weighted variance of alpha-carbon coordinates along a principal component, we use relative hydrophobicity, reversed relative surface accessibility and three (weighted and unweighted) centrality measures described in the paper as weights for the amino acids. In general, a weighted variance is calculated as:

$$s^2 = \frac{1}{n-1}\sum_{i=1}^{n} w_i(x_i - \bar{\mu})^2 \qquad \text{(Eq. S1)}$$

where $w_i$ is the weight (importance) of $i^{\text{th}}$ residue. In this study we took into account the following values as weights: relative hydrophobicity, reverse of the relative surface accessibility, (weighted/unweighted) node degree, (weighted/unweighted) node closeness and (weighted/unweighted) node betweenness.

*Hopkins statistic*

Hopkins statistic [3] shows tendency of a set of (possibly multi-dimensional) points to be clustered. To calculate Hopkins statistic, given a set $X$ of $n$ points (in our case alpha-carbon coordinates) a random sample of size $m \ll n$ is selected. Then a set $Y$ of $m$ uniformly distributed points is generated. For each point in $Y$, two distance measures are defined: $u_i$ is the distance of $y_i$ $(1 \leq i \leq m)$ in $Y$ from its nearest neighbor in $X$, and $w_i$ the distance of a randomly picked point $x_i$ $(1 \leq i \leq m)$ in $X$ from its nearest neighbor in $X$. Hopkins statistic is computed as follows:

$$H = \frac{\sum_{i=1}^{m} u_i}{\sum_{i=1}^{m} u_i + \sum_{i=1}^{m} w_i} \qquad \text{(Eq. S2)}$$

Due to randomness of the Hopkins statistic we repeat the test for 100 times and consider the average of 100 scores as the input feature of the classifier.

*Dip statistic*

Distinguishing whether a set of points (in our case alpha-carbons) possess a unimodal or multimodal distribution can help us to assess clustering tendency of the points. Hartigan's dip statistic [4] was proposed to measure unimodality of a set of points in one-dimensional space. More precisely, dip statistic shows deviation of a given distribution from a reference (unimodal) distribution. So, the lower the dip statistic, the more likely it is that the given distribution is unimodal. We considered dip statistic along each principal component of alpha-carbon coordinates as input features for the single/multi-domain classifier. To construct a one-dimensional distribution over each principal component of alpha-carbon coordinates, a normalized histogram with the bin size of 4 Å is calculated.

*Clustering coefficient*

A triplet in an undirected graph is defined as three nodes that are connected by two (open triplet) or three (closed triplet) edges. So the global clustering coefficient [5] is calculated as:

$$C = \frac{number\ of\ closed\ triplets}{number\ of\ all\ triplets\ (open\ and\ closes)} \qquad \text{(Eq. S3)}$$

Also, the clustering coefficient for the $i^{\text{th}}$ node (local clustering coefficient) [6] in a graph is calculated as:

$$C_i = \frac{2\left|\left\{e_{jk}: v_j, v_k \in N_i, e_{jk} \in E\right\}\right|}{k_i(k_i - 1)} \qquad \text{(Eq. S4)}$$

where for the node $v_i$ the neighborhood $N_i$ is defined as its directly connected neighbor nodes, and $k_i$ is defined as the number of nodes in $N_i$ (i.e. $|N_i|$). The average clustering coefficient is then measured as:

$$\bar{C} = \frac{1}{n}\sum_{i=1}^{n} C_i \qquad \text{(Eq. S5)}$$

*Eigenvalues of the Laplacian matrix*

One of the ways to assess clustering tendency of a graph, is to measure its connectivity strength. Strongly connected graphs have a less tendency to be clustered into subgraphs. Smallest eigenvalues of the Laplacian matrix of a graph are appropriate measures to represent graph connectivity. Since the smallest eigenvalue of a Laplacian matrix is always zero we ignored it. The magnitude of the second-smallest eigenvalue of the Laplacian matrix (known as Fiedler value) of a graph reflects how well the graph can be partitioned into two disjoint subgraphs (how well connected the graph is). In general, the $k$-smallest eigenvectors of the Laplacian matrix can be used to find a useful $k$-way partitioning [7]. Thus, we use the first 10 non-zero smallest eigenvalues ($2^{\text{nd}}$ to $11^{\text{th}}$ eigenvalues) of the normalized Laplacian matrix as input features for the single/multi-domain classifier.

**Table S2:** The list of input features for the single/multi-domain classifier

| # | Feature name | Description |
|---|---|---|
| 1 | n | Protein size in terms of the number of amino acids |
| 2 | e | The number of edges in the protein graph |
| 3 | w_e | Sum of edge weights in the protein graph |
| 4 | e_n_ratio | The number of edges divided by the number of nodes in the protein graph |
| 5 | w_e_n_ratio | Sum of edge weights divided by the number of nodes in the protein graph |
| 6 | radius_of_gyration | The radius of gyration of the protein structure |
| 7 | dip_stat_pc1 | Dip statistic along the 1$^{st}$ principal component of alpha-carbon coordinates |
| 8 | dip_stat_pc2 | Dip statistic along the 2$^{nd}$ principal component of alpha-carbon coordinates |
| 9 | dip_stat_pc3 | Dip statistic along the 3$^{rd}$ principal component of alpha-carbon coordinates |
| 10 | hopkins_stat | Hopkin statistic over alpha-carbon coordinates |
| 11 | clust_coef_g | Global clustering coefficient in the protein graph |
| 12 | clust_coef_al | Average clustering coefficient in the protein graph |
| 13 | clust_coef_al_w | Weighted average clustering coefficient in the protein graph |
| 14 | ca_var_pc1 | Variance along the 1$^{st}$ principal component of alpha-carbon coordinates |
| 15 | ca_var_pc2 | Variance along the 2$^{nd}$ principal component of alpha-carbon coordinates |
| 16 | ca_var_pc3 | Variance along the 3$^{rd}$ principal component of alpha-carbon coordinates |
| 17 | ca_w_var_deg_pc1 | Weighted variance along the 1$^{st}$ principal component of alpha-carbon coordinates by considering node degrees in the protein graph as amino acid weights |
| 18 | ca_w_var_deg_pc2 | Weighted variance along the 2$^{st}$ principal component of alpha-carbon coordinates by considering node degrees in the protein graph as amino acid weights |
| 19 | ca_w_var_deg_pc3 | Weighted variance along the 3$^{rd}$ principal component of alpha-carbon coordinates by considering node degrees in the protein graph as amino acid weights |
| 20 | ca_w_var_w_deg_pc1 | Weighted variance along the 1$^{st}$ principal component of alpha-carbon coordinates by considering weighted node degrees in the protein graph as amino acid weights |
| 21 | ca_w_var_w_deg_pc2 | Weighted variance along the 2$^{st}$ principal component of alpha-carbon coordinates by considering weighted node degrees in the protein graph as amino acid weights |
| 22 | ca_w_var_w_deg_pc3 | Weighted variance along the 3$^{rd}$ principal component of alpha-carbon coordinates by considering weighted node degrees in the protein graph as amino acid weights |
| 23 | ca_w_var_cls_pc1 | Weighted variance along the 1$^{st}$ principal component of alpha-carbon coordinates by considering the closeness of the nodes in the protein graph as amino acid weights |
| 24 | ca_w_var_cls_pc2 | Weighted variance along the 2$^{st}$ principal component of alpha-carbon coordinates by considering the closeness of the nodes in the protein graph as amino acid weights |
| 25 | ca_w_var_cls_pc3 | Weighted variance along the 3$^{rd}$ principal component of alpha-carbon coordinates by considering the closeness of the nodes in the protein graph as amino acid weights |
| 26 | ca_w_var_w_cls_pc1 | Weighted variance along the 1$^{st}$ principal component of alpha-carbon coordinates by considering the weighted closeness of nodes in the protein graph as amino acid weight |
| 27 | ca_w_var_w_cls_pc2 | Weighted variance along the 2$^{st}$ principal component of alpha-carbon coordinates by considering the weighted closeness of nodes in the protein graph as amino acid weight |
| 28 | ca_w_var_w_cls_pc3 | Weighted variance along the 3$^{rd}$ principal component of alpha-carbon coordinates by considering the weighted closeness of nodes in the protein graph as amino acid weight |

| | | |
|---|---|---|
| 29 | ca_w_var_btw_pc1 | Weighted variance along the 1st principal component of alpha-carbon coordinates by considering the betweenness of the nodes in the protein graph as amino acid weights |
| 30 | ca_w_var_btw_pc2 | Weighted variance along the 2st principal component of alpha-carbon coordinates by considering the betweenness of the nodes in the protein graph as amino acid weights |
| 31 | ca_w_var_btw_pc3 | Weighted variance along the 3rd principal component of alpha-carbon coordinates by considering the betweenness of the nodes in the protein graph as amino acid weights |
| 32 | ca_w_var_w_btw_pc1 | Weighted variance along the 1st principal component of alpha-carbon coordinates by considering the weighted betweenness of the nodes in the protein graph as amino acid weights |
| 33 | ca_w_var_w_btw_pc2 | Weighted variance along the 2st principal component of alpha-carbon coordinates by considering the weighted betweenness of the nodes in the protein graph as amino acid weights |
| 34 | ca_w_var_w_btw_pc3 | Weighted variance along the 3rd principal component of alpha-carbon coordinates by considering the weighted betweenness of the nodes in the protein graph as amino acid weights |
| 35 | ca_w_var_acc_pc1 | Weighted variance along the 1st principal component of alpha-carbon coordinates assuming one minus the relative accessible surface of each amino acid as its weight |
| 36 | ca_w_var_acc_pc2 | Weighted variance along the 2nd principal component of alpha-carbon coordinates assuming one minus the relative accessible surface of each amino acid as its weight |
| 37 | ca_w_var_acc_pc3 | Weighted variance along the 3rd principal component of alpha-carbon coordinates assuming one minus the relative accessible surface of each amino acid as its weight |
| 38 | ca_w_var_hydphob_pc1 | Weighted variance along the 1st principal component of alpha-carbon coordinates assuming the relative hydrophobicity of each amino acid as its weight |
| 39 | ca_w_var_hydphob_pc2 | Weighted variance along the 2nd principal component of alpha-carbon coordinates assuming the relative hydrophobicity of each amino acid as its weight |
| 40 | ca_w_var_hydphob_pc3 | Weighted variance along the 3rd principal component of alpha-carbon coordinates assuming the relative hydrophobicity of each amino acid as its weight |
| 41 | deg_mean | Mean degree in the protein graph |
| 42 | deg_var | Degree variance in the protein graph |
| 43 | w_deg_mean | Mean weighted degree in the protein graph |
| 44 | w_deg_var | Weighted degree variance in the protein graph |
| 45 | cls_mean | Mean closeness |
| 46 | cls_var | Closeness variance |
| 47 | w_cls_mean | Mean weighted closeness |
| 48 | w_cls_var | Weighted closeness variance |
| 49 | btw_mean | Mean betweenness |
| 50 | btw_var | Betweenness variance |
| 51 | w_btw_mean | Mean weighted betweenness |
| 52 | w_btw_var | Weighted betweenness variance |
| 53 | acc_mean | Mean accessible surface area |
| 54 | acc_var | Accessible surface area variance |
| 55 | hydphob_mean | Mean hydrophobicity |
| 56 | hydphob_var | Hydrophobicity variance |
| 57 | deg_hydphob_corr | Pearson correlation between degree in the protein graph and amino acid hydrophobicity |
| 58 | w_deg_hydphob_corr | Pearson correlation between weighted degree in the protein graph and amino acid hydrophobicity |
| 59 | deg_acc_corr | Pearson correlation between degree in the protein graph and amino acid accessible surface |

| | | |
|---|---|---|
| 60 | w_deg_hydphob_corr | Pearson correlation between weighted degree in the protein graph and amino acid accessible surface |
| 61 | cls_hydphob_corr | Pearson correlation between closeness in the protein graph and amino acid hydrophobicity |
| 62 | w_cls_hydphob_corr | Pearson correlation between weighted degree in the protein graph and amino acid hydrophobicity |
| 63 | cls_acc_corr | Pearson correlation between closeness in the protein graph and amino acid accessible surface |
| 64 | w_cls_acc_corr | Pearson correlation between weighted closeness in the protein graph and amino acid accessible surface |
| 65 | btw_hydphob_corr | Pearson correlation between betweenness in the protein graph and amino acid hydrophobicity |
| 66 | w_btw_hydphob_corr | Pearson correlation between weighted betweenness in the protein graph and amino acid hydrophobicity |
| 67 | btw_acc_corr | Pearson correlation between betweenness in the protein graph and amino acid accessible surface |
| 68 | w_btw_acc_corr | Pearson correlation between weighted betweenness in the protein graph and amino acid accessible surface |
| 69 | lp_eigval2 | $2^{nd}$-smallest eigenvalue of the protein graph Laplacian matrix (the Fiedler value) |
| 70 | lp_eigval3 | $3^{rd}$-smallest eigenvalue of the protein graph Laplacian matrix |
| 71 | lp_eigval4 | $4^{th}$-smallest eigenvalue of the protein graph Laplacian matrix |
| 72 | lp_eigval5 | $5^{th}$-smallest eigenvalue of the protein graph Laplacian matrix |
| 73 | lp_eigval6 | $6^{th}$-smallest eigenvalue of the protein graph Laplacian matrix |
| 74 | lp_eigval7 | $7^{th}$-smallest eigenvalue of the protein graph Laplacian matrix |
| 75 | lp_eigval8 | $8^{th}$-smallest eigenvalue of the protein graph Laplacian matrix |
| 76 | lp_eigval9 | $9^{th}$-smallest eigenvalue of the protein graph Laplacian matrix |
| 77 | lp_eigval10 | $10^{th}$-smallest eigenvalue of the protein graph Laplacian matrix |
| 78 | lp_eigval11 | $11^{th}$-smallest eigenvalue of the protein graph Laplacian matrix |

**Hyper-parameter optimization**

The single/multi-domain classifier is an ensemble of base classifiers each trained on a balanced bootstrap sample. Thus, the classifier poses four main hyper-parameters: (1) base classifier type, (2) bootstrap size, (3) the number of base classifiers, and (4) the resampling procedure performed on each bootstrap. To determine the first hyper-parameter, an 80%-20% train-test split evaluation was performed and as a result, decision tree was chosen as the base estimator type from a set of candidate models (the others were MLP neural network, SVM with RBF kernel, naïve Bayes). Also, the bootstrap size (the second hyper-parameter) was set equal to the size of the training set ($6862 + 4684 = 11546$) to cover all data as much as possible. In order to set the two other hyper-parameters, namely, the number of decision trees and the resampling procedure (to balance bootstrap samples), a grid search over the training set using 5-fold cross-validation was performed. As a result, the combination of 190 decision trees and SMOTE resampling algorithm [8, 9] was selected as the best choice. The search space for the grid search consisted of all combinations of $\{50{,}60, \dots{,}200\}$ as the number of decision trees and the three resampling procedures: SMOTE, SMOTETomek and TomekLinks..

**Classifier performance**

Although the single/multi-domain classifier was not the main focus of this study, but it exhibited a significant effect on the performance of the overall pipeline. Figure S2 shows the performance of the classifier based on the three measures: accuracy, Matthews correlation coefficient (MCC) [10] and area under ROC curve (AUC). To calculate the performance measures in the test phase we used a different

approach with that of the training phase. If a protein is predicted as single-domain and its main label (according to Table S1) is S or SM, we considered the prediction as true. As the same way, if a protein is predicted as multi-class and its main label is M, M! or SM, we considered the prediction as true. By comparing Figure S2 with overall performance of the method (Table 2 and Figure 9 in the main paper), one can notice that most errors of KluDo are due to misclassification of the proteins into single- or multi-domain classes. Also, Figure S3 and Figure S4 show recall, precision and F-measure (F1) for each of the single-domain and multi-domain classes over the test sets. Also, Figure S5 shows ROC curve for each of the datasets by considering multi-domain proteins as positive class.

Since, decision tree was used as the base classifier of the bagging classifier, its interpretability allows us to obtain the degree of importance for each of the features. Figure S6 shows the list of 78 input features of the classifier, sorted by the degree of importance and the degree of importance itself. As seen in the figure, the features that are related to the first principal component of alpha-carbon coordinates are of higher importance in the classification of protein structures into single- or multi-domain classes. Also, the second eigenvalue of the Laplacian matrix that is known as Fiedler value has gained a high degree of importance. It is also true about dip statistic.

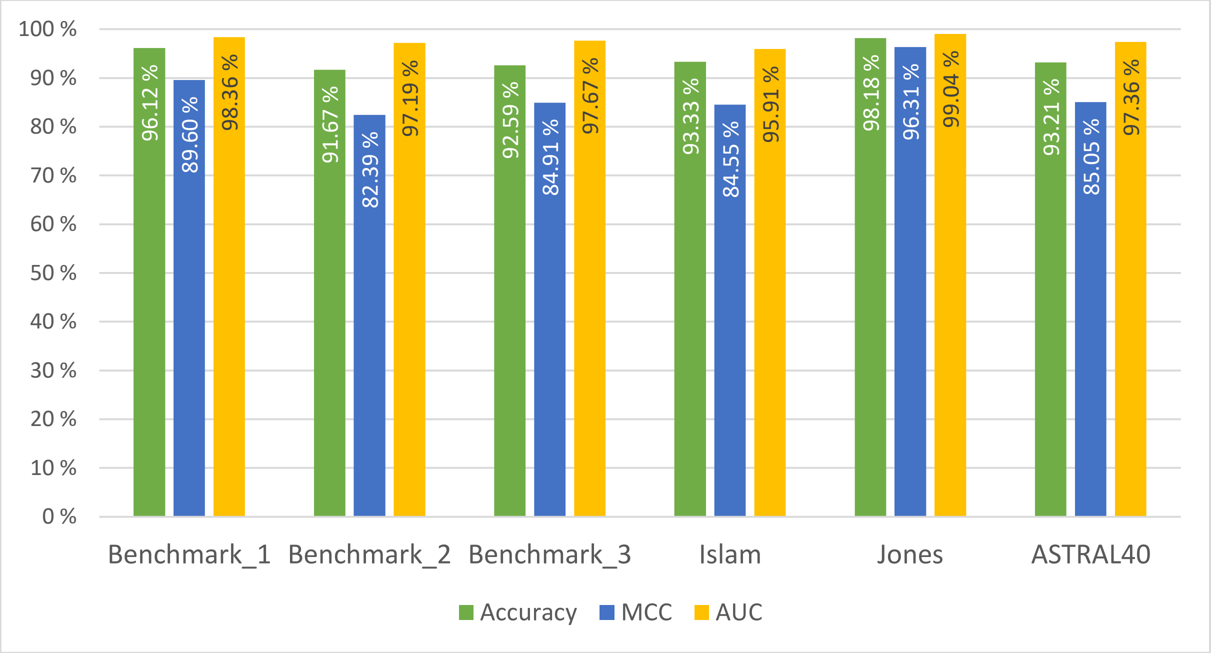


**Figure S2:** General performance of the single/multi-domain classifier over the benchmarks

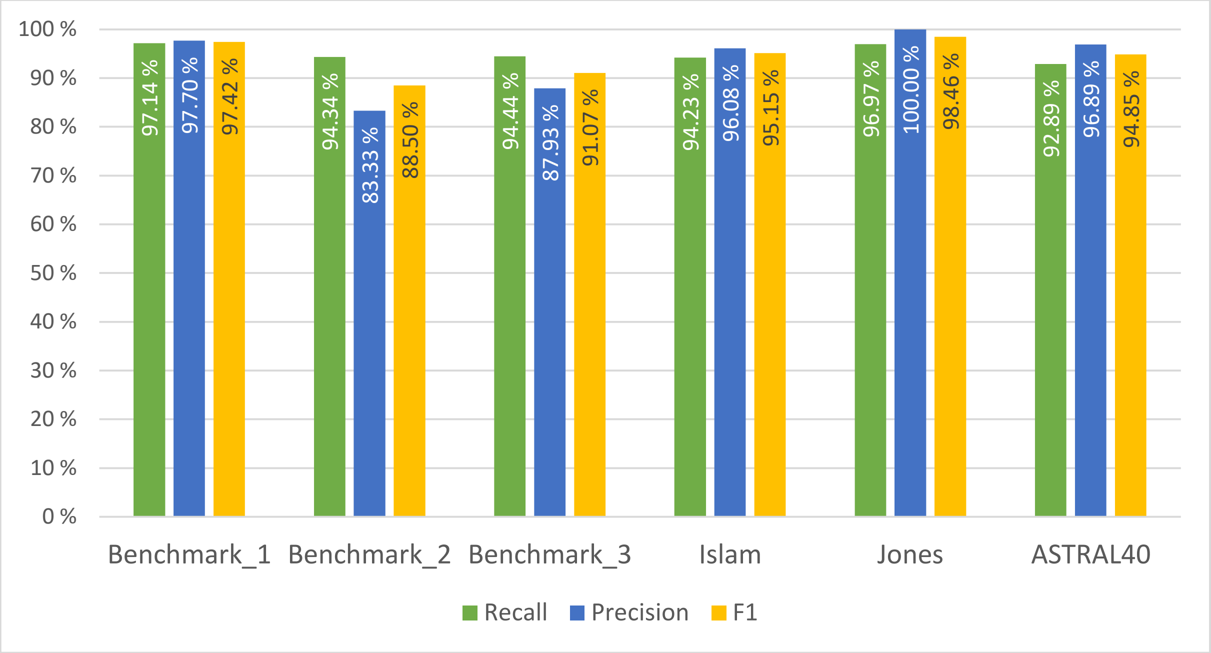


**Figure S3:** Performance of the single/multi-domain classifier over the single-domain proteins

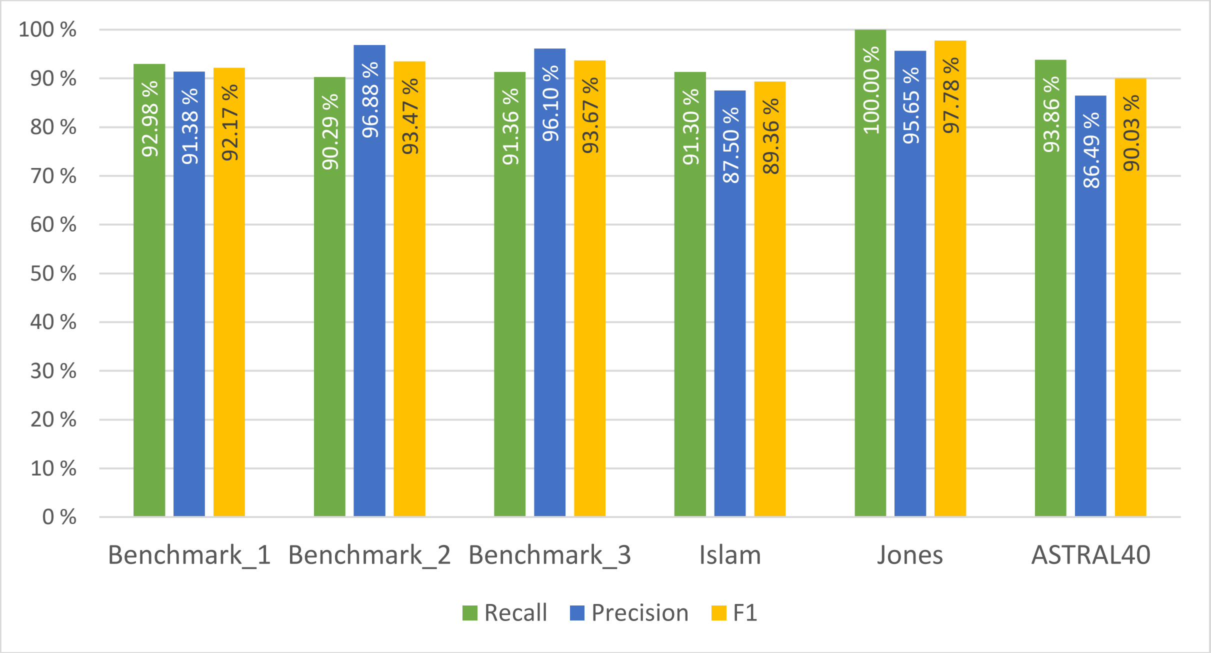


**Figure S4:** Performance of the single/multi-domain classifier over the multi-domain proteins

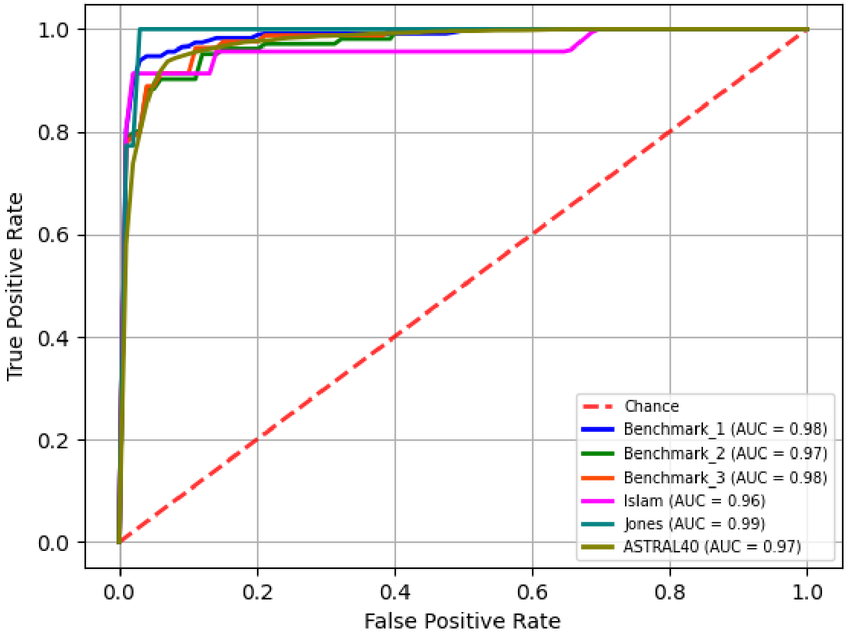


**Figure S5:** ROC curve for each of the datasets by considering multi-domain proteins as positive class.

## Bandwidth determination

In this study the bandwidth value for each kernel function is calculated as follows:

$$bw = \eta . {R_g}^2 \qquad \text{(Eq. S6)}$$

where $R_g$ is the radius of gyration of the protein structure and $\eta$ is a coefficient that is determined for each kernel. We inspected the performance of all combinations of kernels and clustering methods by running KluDo on multi-domain structures of the training set (MM category in Table S1) over predefined sequences of values for $\eta$, while assuming all structures as multi-domain (single/multi-domain classifier was not used). For each kernel a distinct range of values were used for $\eta$. Figure S7 shows the accuracies based on OL score with the threshold of 85% for different values of $\eta$. The accuracies are calculated after removing protein chains with fixed OL score across all values of $\eta$.

Since in most cases there is no a clear peak point in the curves, we decided to choose three values of $\eta$ with highest accuracies instead of choosing a single value. The selected values for are indicated in Figure S6 with the dotted lines. Table S3 shows the accuracy of KluDo over the multi-domain structures of ASTRAL40 (based on MM category condition) for each of the three values of $\eta$ when using all kernel function- clustering method pairs. For each case two accuracies based on OL and ARI (both with the threshold of 85%) scores are reported. Highlighted cells indicate the best cases on which results are reported in the main text.

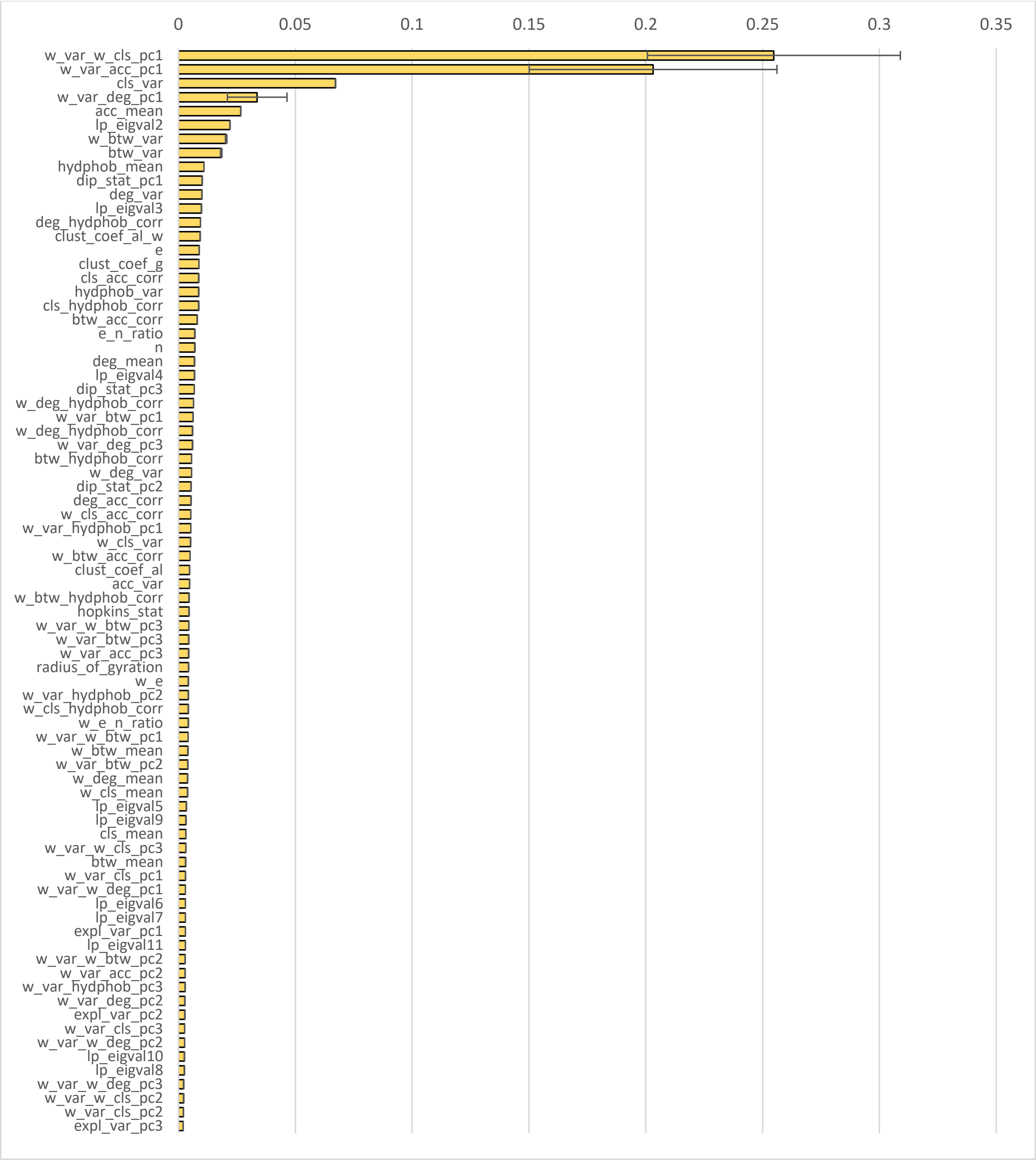


**Figure S6:** Feature importance by interpreting the single/multi-domain classifier

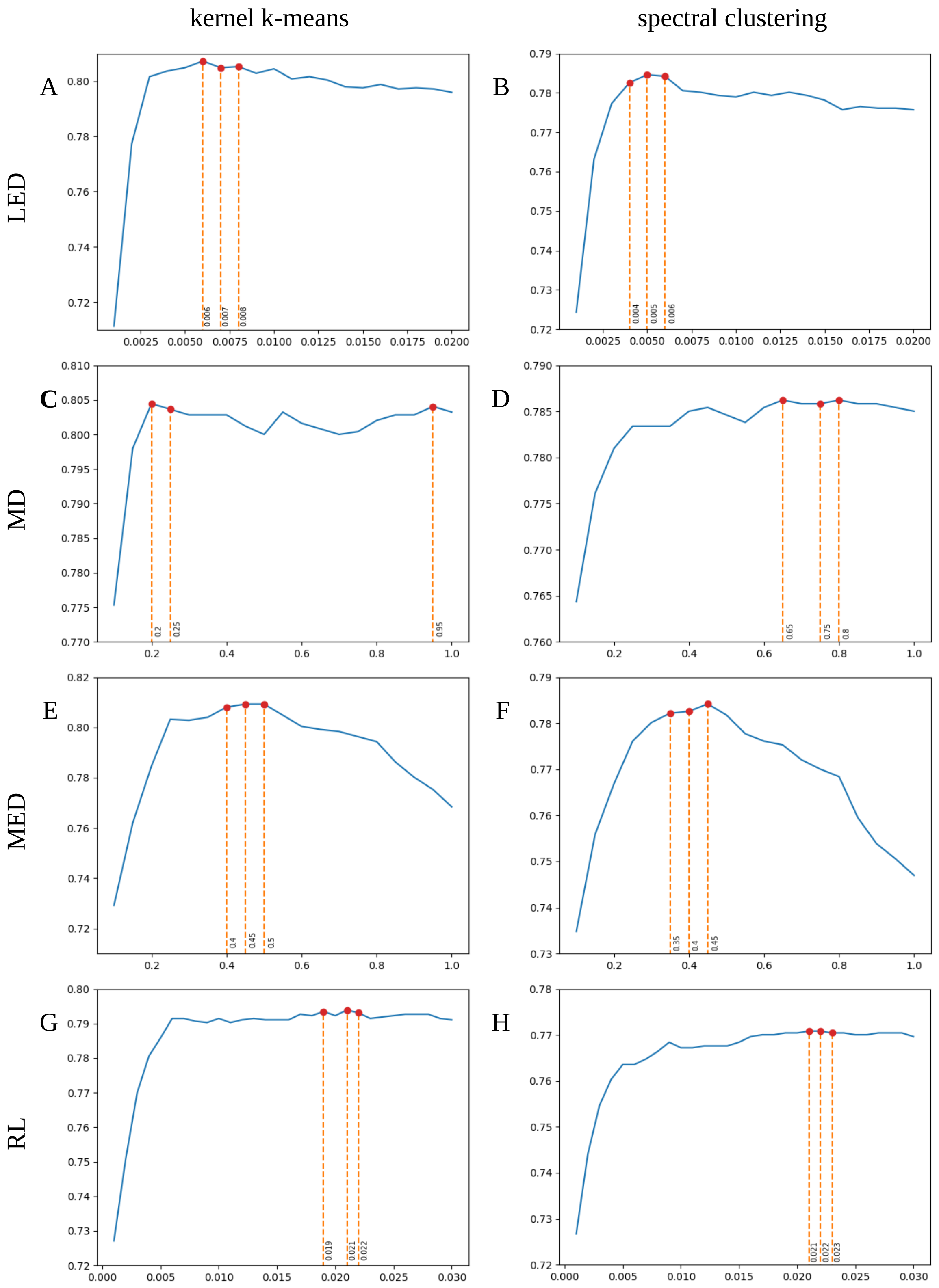


**Figure S7:** The accuracies (based in OL score with the threshold of 85%) for different values of $\eta$ over multi-domain structures of the training set. The plots show the results for the kernels LED (**A** and **B**), MD (**C** and **D**), MED (**E** and **F**) and RL (**G** and **H**) when using kernel k-means (**A**, **C**, **E** and **G**) and spectral clustering (**B**, **D**, **F** and **H**) algorithms. The three highest accuracy values of $\eta$ are also determined by dotted lines.

**Table S3:** KluDo's performance over the multi-domain structures of ASTRAL40, with multi-domain assumption, based on the three candidate values of $\eta$ for each kernel function-clustering method. The accuracies are based on 85% threshold for the overlapping score (OL) and ARI. KK and SP stand for kernel k-means and spectral clustering, respectively.

| | | $\eta$ | OL | ARI | $\eta$ | OL | ARI | $\eta$ | OL | ARI |
|---|---|---|---|---|---|---|---|---|---|---|
| **LED** | **KK** | 0.006 | 68.66% | 55.62% | 0.007 | 68.16% | 55.07% | 0.008 | 67.71% | 54.48% |
| | **SP** | 0.004 | 67.30% | 54.89% | 0.005 | 67.26% | 54.71% | 0.006 | 67.12% | 54.62% |
| **MD** | **KK** | 0.2 | 68.57% | 55.93% | 0.25 | 68.89% | 56.30% | 0.95 | 66.62% | 52.85% |
| | **SP** | 0.65 | 66.62% | 54.17% | 0.75 | 66.71% | 54.35% | 0.8 | 66.76% | 54.30% |
| **MED** | **KK** | 0.4 | 69.20% | 56.20% | 0.45 | 68.70% | 55.71% | 0.5 | 68.57% | 55.66% |
| | **SP** | 0.35 | 67.26% | 54.94% | 0.4 | 66.89% | 54.76% | 0.45 | 66.71% | 54.44% |
| **RL** | **KK** | 0.019 | 65.22% | 52.58% | 0.021 | 65.31% | 52.72% | 0.022 | 65.31% | 52.58% |
| | **SP** | 0.021 | 62.77% | 50.77% | 0.022 | 62.82% | 50.77% | 0.023 | 62.73% | 50.72% |

## Randomized graph tests

In order to assess the suitability of graph construction procedure we performed a set of randomization tests. For this purpose, the multi-domain chains (based on MM category definition in Table S1) of the three datasets benchmark_1, benchmark_2 and benchmark_3 were chosen. Two different randomization methods were used. In the first test, for each protein, random weights were assigned to the edges of the original graph topology based on a uniform weight distribution whose minimum and maximum values are obtained from the original protein graph constructed by the standard procedure of KluDo. In the second test, both topology and weights were generated randomly. For each protein, graph topology was generated by random rewiring of the original protein graph such that the degree distribution is preserved, and the weights were assigned randomly by the procedure used in the previous test. All decompositions were done using LED as the kernel function and spectral clustering as the clustering method. Also, it was assumed that all the structures consist of at least two domains since only multi-domain proteins were selected for the analysis.

The mean accuracy across 30 runs for each of the tests was calculated. While the mean accuracy from the first test (randomized weights) showed a significant decrease over Benchmark_2 and Benchmark_3, the mean accuracy from the second test (randomized topology and weights) were about to zero over all three datasets. From these results one can conclude that the graph topology contains much more information about the protein structures than edge weights. Figure S8 shows the accuracy of the standard KluDo procedure along with the mean accuracy resulted from the first test (randomized weights) over the multi-domain proteins in Benchmark_1, Bebchmark_2 and Benchmark_3.

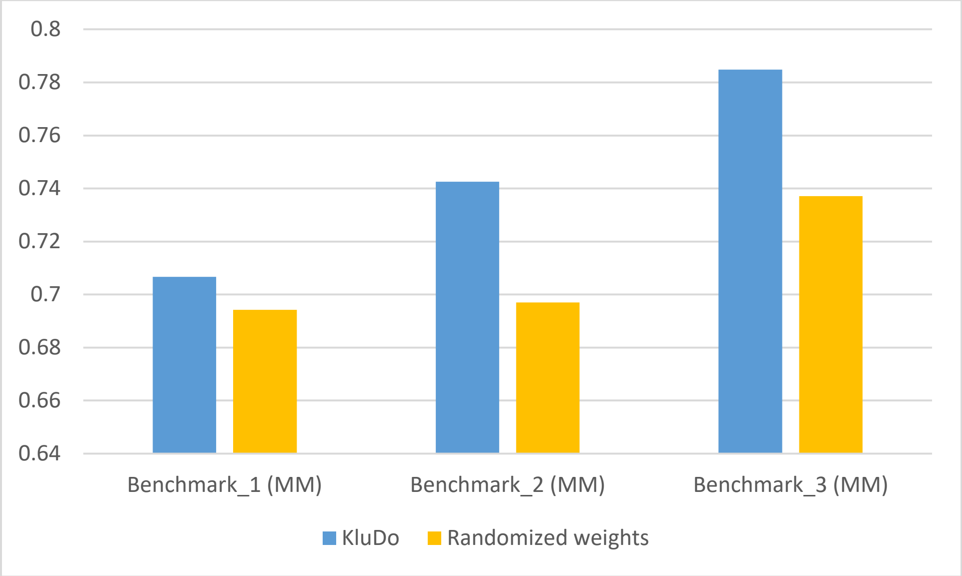


**Figure S8:** Accuracy of the standard KluDo procedure versus the mean accuracy resulted from weight randomization over the multi-domain proteins in Benchmark_1, Bebchmark_2 and Benchmark_3.

## Implementation

The whole project was written in Python 3.7. The Python packages atomium [11] (parsing PDB files), igraph [12] (graph procedures), pyclustertend [13] (Hopkins statistic), unidip [14] (dip statistic), scikit-learn [15] (spectral clustering, atomic contact calculation, single/multi-domain classifier evaluation), imbalanced-learn [16] (scikit-learn wrapper, single/multi-domain classification), tslearn [17] (kernel k-means clustering) and the command-line program DSSP [18, 19] were used in this project. KluDo can be executed as a Windows/Linux/Mac command-line application. We also developed a web application using the Django framework [20] to make KluDo available through the world wide web, so that users can enter a protein chain as well as algorithm-related arguments. Once the algorithm is executed, the result is represented in both text form and 3D structure powered by Jmol [21].

All the parameters in Table 1 besides kernel function, clustering method and lower/upper bound for the number of domains can be set optionally by the users in both command-line and web application. In addition to the algorithm-related arguments, we made it possible for the users to enter a range for the number of domains as an input argument. The source code of this project is available on Github at https://github.com/taherimo/kludo. Also, the web application is accessible from http://cbph.ir/tools/kludo.

## References


[1] A. Bavelas, "Communication patterns in task-oriented groups," *The journal of the acoustical society of America,* vol. 22, no. 6, pp. 725-730, 1950.

[2] L. C. Freeman, "A set of measures of centrality based on betweenness," *Sociometry,* pp. 35-41, 1977.

[3] B. Hopkins and J. G. Skellam, "A new method for determining the type of distribution of plant individuals," *Annals of Botany,* vol. 18, no. 2, pp. 213-227, 1954.

[4] J. A. Hartigan and P. M. Hartigan, "The dip test of unimodality," *The annals of Statistics,* pp. 70-84, 1985.

[5] P. W. Holland and S. Leinhardt, "Transitivity in structural models of small groups," *Comparative group studies,* vol. 2, no. 2, pp. 107-124, 1971.

[6] D. J. Watts and S. H. Strogatz, "Collective dynamics of 'small-world'networks," *nature,* vol. 393, no. 6684, pp. 440-442, 1998.

[7] J. R. Lee, S. O. Gharan and L. Trevisan, "Multiway spectral partitioning and higher-order cheeger inequalities," *Journal of the ACM (JACM),* vol. 61, no. 6, pp. 1-30, 2014.

[8] S. Wang and X. Yao, "Diversity analysis on imbalanced data sets by using ensemble models," in *2009 IEEE symposium on computational intelligence and data mining*, 2009.

[9] N. V. Chawla, K. W. Bowyer, L. O. Hall and W. P. Kegelmeyer, "SMOTE: synthetic minority over-sampling technique," *Journal of artificial intelligence research,* vol. 16, pp. 321-357, 2002.

[10] B. W. Matthews, "Comparison of the predicted and observed secondary structure of T4 phage lysozyme," *Biochimica et Biophysica Acta (BBA)-Protein Structure,* vol. 405, no. 2, pp. 442-451, 1975.

[11] S. M. Ireland and A. C. Martin, "atomium: a python structure parser," *Bioinformatics,* vol. 36, no. 9, pp. 2750-2754, 2020.

[12] G. Csardi and T. Nepusz, "The igraph software package for complex network research," *InterJournal,* vol. Complex Systems, p. 1695, 2006.

[13] I. Lachheb, *pyclustertend,* 2021.

[14] S. Maurus and C. Plant, "Skinny-dip: clustering in a sea of noise," in *Proceedings of the 22nd ACM SIGKDD international conference on Knowledge discovery and data mining,* 2016.

[15] F. Pedregosa, G. Varoquaux, A. Gramfort, V. Michel, B. Thirion, O. Grisel, M. Blondel, P. Prettenhofer, R. Weiss, V. Dubourg, J. Vanderplas, A. Passos, D. Cournapeau, M. Brucher, M.

Perrot and E. Duchesnay, "Scikit-learn: Machine Learning in Python," *Journal of Machine Learning Research,* vol. 12, pp. 2825-2830, 2011.

[16] G. Lemaître, F. Nogueira and C. K. Aridas, "Imbalanced-learn: A Python Toolbox to Tackle the Curse of Imbalanced Datasets in Machine Learning," *Journal of Machine Learning Research,* vol. 18, no. 17, pp. 1-5, 2017.

[17] {. Tavenard, J. Faouzi, G. Vandewiele, F. Divo, G. Androz, C. Holtz, M. Payne, R. Yurchak, M. Rußwurm, K. Kolar and E. Woods, "Tslearn, A Machine Learning Toolkit for Time Series Data," *Journal of Machine Learning Research,* vol. 21, no. 118, pp. 1-6, 2020.

[18] W. Kabsch and C. Sander, "Dictionary of protein secondary structure: pattern recognition of hydrogen-bonded and geometrical features," *Biopolymers: Original Research on Biomolecules,* vol. 22, no. 12, pp. 2577-2637, 1983.

[19] R. P. Joosten, T. A. Te Beek, E. Krieger, M. L. Hekkelman, R. W. Hooft, R. Schneider, C. Sander and G. Vriend, "A series of PDB related databases for everyday needs," *Nucleic acids research,* vol. 39, no. suppl_1, pp. D411-D419, 2010.

[20] Django Software Foundation, *Django,* 2019.

[21] *Jmol: an open-source Java viewer for chemical structures in 3D,* 2008.
